# Correlated electronic structures and unconventional superconductivity in bilayer nickelate heterostructures

**DOI:** 10.1093/nsr/nwaf253

**Published:** 2025-06-23

**Authors:** Changming Yue, Jian-Jian Miao, Haoliang Huang, Yichen Hua, Peng Li, Yueying Li, Guangdi Zhou, Wei Lv, Qishuo Yang, Fan Yang, Hongyi Sun, Yu-Jie Sun, Junhao Lin, Qi-Kun Xue, Zhuoyu Chen, Wei-Qiang Chen

**Affiliations:** State Key Laboratory of Quantum Functional Materials, Department of Physics, and Guangdong Basic Research Center of Excellence for Quantum Science, Southern University of Science and Technology, Shenzhen 518055, China; Quantum Science Center of Guangdong-Hong Kong-Macao Greater Bay Area, Shenzhen 518045, China; Guangdong Provincial Key Laboratory of Advanced Thermoelectric Materials and Device Physics, Southern University of Science and Technology, Shenzhen 518055, China; Quantum Science Center of Guangdong-Hong Kong-Macao Greater Bay Area, Shenzhen 518045, China; State Key Laboratory of Quantum Functional Materials, Department of Physics, and Guangdong Basic Research Center of Excellence for Quantum Science, Southern University of Science and Technology, Shenzhen 518055, China; State Key Laboratory of Quantum Functional Materials, Department of Physics, and Guangdong Basic Research Center of Excellence for Quantum Science, Southern University of Science and Technology, Shenzhen 518055, China; Quantum Science Center of Guangdong-Hong Kong-Macao Greater Bay Area, Shenzhen 518045, China; State Key Laboratory of Quantum Functional Materials, Department of Physics, and Guangdong Basic Research Center of Excellence for Quantum Science, Southern University of Science and Technology, Shenzhen 518055, China; State Key Laboratory of Quantum Functional Materials, Department of Physics, and Guangdong Basic Research Center of Excellence for Quantum Science, Southern University of Science and Technology, Shenzhen 518055, China; Quantum Science Center of Guangdong-Hong Kong-Macao Greater Bay Area, Shenzhen 518045, China; State Key Laboratory of Quantum Functional Materials, Department of Physics, and Guangdong Basic Research Center of Excellence for Quantum Science, Southern University of Science and Technology, Shenzhen 518055, China; State Key Laboratory of Quantum Functional Materials, Department of Physics, and Guangdong Basic Research Center of Excellence for Quantum Science, Southern University of Science and Technology, Shenzhen 518055, China; State Key Laboratory of Quantum Functional Materials, Department of Physics, and Guangdong Basic Research Center of Excellence for Quantum Science, Southern University of Science and Technology, Shenzhen 518055, China; State Key Laboratory of Quantum Functional Materials, Department of Physics, and Guangdong Basic Research Center of Excellence for Quantum Science, Southern University of Science and Technology, Shenzhen 518055, China; School of Physics, Beijing Institute of Technology, Beijing 100081, China; Shenzhen Institute for Quantum Science and Engineering, Southern University of Science and Technology, Shenzhen 518055, China; State Key Laboratory of Quantum Functional Materials, Department of Physics, and Guangdong Basic Research Center of Excellence for Quantum Science, Southern University of Science and Technology, Shenzhen 518055, China; Quantum Science Center of Guangdong-Hong Kong-Macao Greater Bay Area, Shenzhen 518045, China; State Key Laboratory of Quantum Functional Materials, Department of Physics, and Guangdong Basic Research Center of Excellence for Quantum Science, Southern University of Science and Technology, Shenzhen 518055, China; Quantum Science Center of Guangdong-Hong Kong-Macao Greater Bay Area, Shenzhen 518045, China; Guangdong Provincial Key Laboratory of Advanced Thermoelectric Materials and Device Physics, Southern University of Science and Technology, Shenzhen 518055, China; State Key Laboratory of Quantum Functional Materials, Department of Physics, and Guangdong Basic Research Center of Excellence for Quantum Science, Southern University of Science and Technology, Shenzhen 518055, China; Quantum Science Center of Guangdong-Hong Kong-Macao Greater Bay Area, Shenzhen 518045, China; Department of Physics, Tsinghua University, Beijing 100084, China; State Key Laboratory of Quantum Functional Materials, Department of Physics, and Guangdong Basic Research Center of Excellence for Quantum Science, Southern University of Science and Technology, Shenzhen 518055, China; Quantum Science Center of Guangdong-Hong Kong-Macao Greater Bay Area, Shenzhen 518045, China; State Key Laboratory of Quantum Functional Materials, Department of Physics, and Guangdong Basic Research Center of Excellence for Quantum Science, Southern University of Science and Technology, Shenzhen 518055, China; Quantum Science Center of Guangdong-Hong Kong-Macao Greater Bay Area, Shenzhen 518045, China

**Keywords:** epitaxial thin film, La_3_Ni_2_O_7_, cluster dynamical mean-field theory, correlated electronic structure, unconventional pairing mechanism

## Abstract

The recent discovery of ambient-pressure superconductivity in thin-film bilayer nickelates opens new possibilities for investigating electronic structures in this new class of high-transition-temperature ($T_\mathrm{c}$) superconductors. Here, we construct a realistic multi-orbital Hubbard model for the thin-film system based on structural parameters integrating scanning transmission electron microscopy measurements and *ab initio* calculations. The interaction parameters are calculated with the constrained random phase approximation (cRPA). Density functional theory (DFT) plus cluster dynamical mean-field theory (CDMFT) calculations, with cRPA-calculated on-site Coulomb repulsive $U$ and experimentally measured electron filling $n$, quantitatively reproduce Fermi surfaces from angle-resolved photoemission spectroscopy experiments. The distinct Fermi surface topology from simple DFT+$U$ results features the indispensable role of correlation effects. Based upon the correlated electronic structures, a modified random-phase-approximation (RPA) approach yields a pronounced $s^{\pm }$-wave pairing instability, due to the strong spin fluctuations originating from the Fermi surface nesting between bands with predominantly $d_{z^{2}}$ characters. Our findings highlight the quantitative effectiveness of the DFT+cRPA+CDMFT approach that precisely determines correlated electronic structure parameters without fine-tuning. The revealed intermediate correlation effect may explain the same order-of-magnitude onset $T_\mathrm{c}$ observed both in pressured bulk and strained thin-film bilayer nickelates.

## Teaser

This study shows that non-local interlayer correlations lead to correlated electronic structures and Fermi surface reconstruction, triggering unconventional s±-wave superconductivity in Ruddlesden-Popper bilayer nickelate thin films.

## INTRODUCTION

The high-temperature superconductivity has been a key area of research in condensed matter physics [1–3]. In recent years, the high-temperature superconductivity under pressure in Rudlesden-Popper nickelates has garnered significant attention [4]. Extensive experimental [5–15] and theoretical [14–35] studies have been conducted to investigate their physical properties and superconducting mechanisms. The high-pressure conditions required for the superconducting phase in bulk compounds pose significant challenges for detailed property measurements [9]. Recently, the discovery of ambient-pressure superconductivity in bilayer nickelate epitaxial thin films grown on SrLaAlO$_4$ substrates opens new possibilities for experimental investigations [36–38]. Structurally, the out-of-plane (i.e. $c$ axis) lattice constants in compressively strained films are elongated, opposite to anisotropically pressured bulks despite their similar onset $T_\mathrm{C}$, raising an intriguing question about the role of the inter-layer coupling via the $d_{z^{2}}$ orbital in superconductivity.

On the theoretical front, understanding the electronic structure is essential for exploring the superconducting mechanism of these materials. There has been considerable debate on the electronic structure and the role played by the correlation effect in superconducting bilayer nickelate bulks under high pressure [8,15,19,24,29,33,39]. The superconducting bilayer nickelate thin films under ambient pressure enable angle-resolved photoemission spectroscopy (ARPES) experiments that serve as a benchmark to address this issue [40]. In this work, we study the correlated electronic structure and the pairing mechanism of bilayer nickelate superconducting thin films, based on structural parameters from experiments, and achieve quantitative agreements with ARPES Fermi surfaces without parameter fine-tuning.

## RESULTS

### Experimental structural analysis

**Figure 1.**
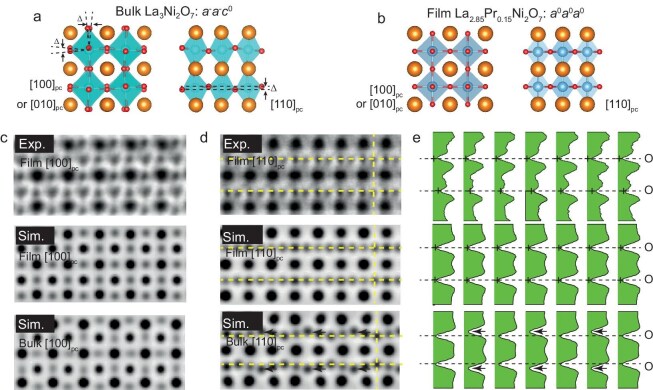
Oxygen octahedral rotation analysis in the La$_{2.85}$Pr$_{0.15}$Ni$_2$O$_7$ film. (a) Schematic crystal structure of the bulk La$_3$Ni$_2$O$_7$ with an $a^-a^-c^0$ oxygen octahedral rotation pattern. Displacements of oxygen atoms are indicated by $\Delta$. (b) Schematic crystal structure of the La$_{2.85}$Pr$_{0.15}$Ni$_2$O$_7$ film grown on an SrLaAlO$_4$ substrate with an $a^0a^0a^0$ oxygen octahedral rotation pattern, showing no splitting or displacement in any direction. (c, d) Enlarged ABF images of the cross section of the La$_{2.85}$Pr$_{0.15}$Ni$_2$O$_7$ film, projected along [100]$_{pc}$ and [110]$_{pc}$, respectively. ABF images ([100]$_{pc}$, [010]$_{pc}$ and [110]$_{pc}$) with larger fields of view are shown in Fig. S1. From top to bottom are the experimental results of the film, the simulation results of the film and the simulation results of the bulk. (e) The corresponding O atom column intensity profile on the vertical dashed line in the experimental and simulation results in panel (d). Only a column of O atoms on the right is annotated with dashed lines in panel (d). Crosses indicate the positions of the local minima corresponding to the positions of oxygen atoms. Dashed lines mark the same positions as the horizontal dashed lines in panel (d). Black arrows indicate the positions of the oxygen atoms deviating from the dashed lines.

We begin with scanning transmission electron microscopy (STEM) analysis of oxygen octahedral rotations in a superconducting La$_{2.85}$Pr$_{0.15}$Ni$_2$O$_7$ epitaxial film grown on an SrLaALO$_4$ substrate [37]. In ambient-pressure bulk La$_3$Ni$_2$O$_7$ crystals, the $a^-a^-c^0$ oxygen octahedral rotation pattern is generally seen (Fig. 1a): the NiO$_6$ octahedra undergo inverse rotations along the $a$ and $b$ axes (i.e. [100]$_{pc}$ and [010]$_{pc}$), while no rotation occurs along the $c$ axis ([001]$_{pc}$). Note that [100]$_{pc}$ and [010]$_{pc}$ are symmetric directions under the symmetry group. This rotation leads to splittings of oxygen positions along the [100]$_{pc}$/[010]$_{pc}$ and [110]$_{pc}$ projections. In contrast, the La$_{2.85}$Pr$_{0.15}$Ni$_2$O$_7$ film grown on SrLaAlO$_4$ appears more likely to conform to the high-symmetry $a^0a^0a^0$ pattern (Fig. 1b). This pattern features the absence of splittings of the oxygen positions in any projection directions. Panels (c) and (d) of Fig. 1 present enlarged annular bright-field (ABF) images of the La$_{2.85}$Pr$_{0.15}$Ni$_2$O$_7$ film cross section, projected along the [100]$_{pc}$ and [110]$_{pc}$ directions, respectively (see Fig. S1 for larger field-of-view images). In the [100]$_{pc}$ direction, the oxygen atoms in both the experimental and simulated film results appear rounder compared to the elliptical shape observed in the simulated bulk. In the [110]$_{pc}$ direction, the oxygen atoms exhibit a distinct zigzag feature in the Ni-O layer for the bulk simulated case, which is not present in the film measurement and the film simulation. The line profiles of oxygen atoms (Fig. 1e) more clearly demonstrate their alignment, in contrast to the bulk simulations. These results suggest that the coherent epitaxy may suppress the oxygen octahedral rotations in the film, giving rise to a higher symmetry of the lattice, thus further altering orbital overlap and bonding interactions between Ni and O atoms. Detailed positions of each cation in the lattice are measured by combining STEM and X-ray diffraction (XRD) experiments, as shown in Table 1. Note that the structure and atomic positions of the film may vary with the change of the rare earth elements and the existence of oxygen vacancies.

**Table 1.** The lattice parameters of the La$_{2.85}$Pr$_{0.15}$Ni$_2$O$_7$ film on substrate SrLaAlO$_4$. STEM measurements are calibrated by the lattice constants obtained from X-ray diffraction experiments. The term ‘La-La intra.’ refers to the distance between La atoms in the $c$-axis direction within the perovskite layer, while ‘La-La inter.’ refers to the distance between adjacent La atoms in the $c$-axis direction between the perovskite bilayers. The abbreviation ‘sub.’, standing for substrate, is used here to save space.

	SrLaAlO$_4$			La$_{2.85}$Pr$_{0.15}$Ni$_2$O$_7$	
	cif (sub.)	XRD (sub.)	STEM (sub.)	XRD (film)	STEM (film)
$a$ (Å)	$\hphantom{0}3.7544$	$\hphantom{0}3.754$	–	$\hphantom{0}3.754$	–
$b$ (Å)	$\hphantom{0}3.7544$	$\hphantom{0}3.754$	–	$\hphantom{0}3.754$	–
$c$ (Å)	$12.649\hphantom{0}$	12.634	–	20.819	–
Ni-O-Ni angle (deg)		–	–	–	$180\pm 5$
Ni-Ni length (Å)		–	–	–	$4.28\pm 0.05$
La-La intra. (Å)		–	$3.61\pm 0.05$	–	$3.71\pm 0.05$
La-La inter. (Å)		–	$2.69\pm 0.05$	–	$3.0\pm 0.05$

### *Ab initio* calculations

**Figure 2.**
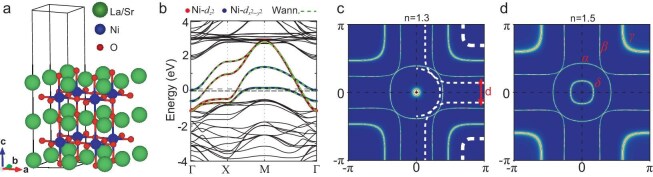
Crystal structure and DFT+$U$ results ($U=5$ eV, $J=1$ eV). (a) The half-UC thin-film crystal structure of La$_3$Ni$_2$O$_7$ constructed using structural parameters from the thin film of La$_{2.85}$Pr$_{0.15}$Ni$_2$O$_7$. (b) The DFT+$U$ bands (black solid line) and their Wannier interpolation (green dashed line) of the half-UC thin film. The thin (thick) dashed line marks the Fermi energy for the system with (without) Sr doping. The size of the red (blue) dots demonstrates the projected weight for Ni-$d_{z^2}$ (Ni-$d_{x^2-y^2}$) orbitals. (c, d) Fermi surfaces of the Wannier TB model at $n=1.3$ and $n=1.5$ per Ni site, respectively. Names of all electron or hole pockets are labeled in (d). In panel (c), the extracted ARPES Fermi surface [40] is overlaid as the white dashed line. Only the right half is shown for clarity.

The crystal structure of thin films is constructed for DFT+$U$ calculations, using realistic structural parameters (Table 1). A simple yet effective choice is to use the half unit cell (UC) containing one nickelate bilayer (space group *P*4/*mmm*; see Fig. 2a), constructed by slicing the high-pressure Fmmm bulk structure. Experiments show that Sr substitution of La near the interface due to interfacial diffusion from the substrate [37] may introduce moderate hole doping into the system. Such an Sr-doping effect is simulated by the virtual crystal approximation, and the resultant band structure can be approximated by the rigid-band shift of the Fermi level in the system without Sr. In the structural relaxation within DFT (Perdew-Burke-Ernzerhof (PBE) functional), only the oxygen atoms are relaxed along the $c$ axis, while the distances between heavy atoms like La and Ni are fixed to their STEM values (see Table 1). In particular, the inter-layer Ni-Ni distance is fixed to 4.28 Å, which is larger than its fully relaxed value 3.97 Å. The large discrepancy in the inter-layer Ni-Ni distances between the PBE-based free relaxation and STEM measurement will be investigated in the future with the advanced DFT+eDMFT–based structural relaxation, which successfully reproduces the Se height in moderately correlated FeSe [41]. This method calculates forces accounting for the dynamical many-body correlation effects missing in PBE, which significantly underestimate the Se height in FeSe. More details of DFT+$U$ calculations and lattice relaxation can be found in the supplementary material. The band structure is shown in Fig. 2b. Similar to the bulk structure [14,39], the low-energy bands are mainly formed by the Ni-$e_g$ orbitals, as demonstrated by the orbital-projected bands.

A realistic multi-orbital Hubbard model for the Ni-$e_g$ subspace is formulated (see the Methods section below). The first part is the tight-binding model, which has bands aligned well with the DFT+$U$ band (see Fig. 2b). The tight-binding (TB) model Hamiltonian in ${\bf k}$ space has the same matrix structure as in [14]. The on-site energy level $\epsilon _{x/z}$ and the hopping parameters are summarized in Table 2. Here, we introduce the inter-layer bonding ($+$) and anti-bonding ($-$) orbital bases $d_{z_{\pm }}=(d_{Az}\pm d_{Bz})/\sqrt{2}$ and $d_{x_{\pm }}=(d_{Ax}\pm d_{Bx})/\sqrt{2}$ ($A,B$ denote the layer indexes, $x\equiv d_{x^2-y^2}$, $z\equiv d_{z^2}$). Compared to the high-pressure bulk crystal, the thin film has two main differences in the TB model parameters that significantly affect the bands and Fermi surface topology. First, the inter-layer intra-$z$-orbital hopping $t_{\perp ,\text{0.5UC}}^z=-0.439$ eV is nearly 30% (0.2 eV) smaller in magnitude than $t_{\perp ,\text{HP}}^z=-0.635$ eV. Second, the crystal-field splitting $\Delta E_{{0.5{\rm UC}}}=\epsilon _{x}-\epsilon _{z}$ is enhanced to 0.519 eV, roughly $\sim\!\! 40\%$ larger than $\Delta E_{\text{HP}}=0.367$ eV. Smaller $t_{\perp }^z$ means smaller bonding–anti-bonding splitting, which leads to the coexistence of a $\delta$ electron pocket (located at the $\Gamma$ point) formed by the $z_-$ band and the $\gamma$-hole pocket (located at the $M$ point) formed by the $z_+$ band, as shown in panels (c) and (d) of Fig. 2 for both hole-doped (with 0.2 hole doping per Ni) and undoped scenarios. This coexistence, however, does not happen in both high-pressure [14] and ambient-pressure bulk crystals [39].

**Table 2.** The tight-binding parameters (in units of electronvolts) for the half-UC thin film obtained from DFT+$U$. The number in parentheses corresponds to that of the high-pressure bulk structure reported by Luo *et al.* [14].


$t_{1}^{x}$	$-0.466\ (-0.483)$
$t_{1}^{z}$	$-0.126\ (-0.110)$
$t_{2}^{x}$	$0.062\ (0.069)$
$t_{2}^{z}$	$-0.016\ (-0.017)$
$t_{3}^{xz}$	$0.229\ (0.239)$
$t_{\bot }^{x}$	$0.001\ (0.005)$
$t_{\bot }^{z}$	$-0.439\ (-0.635)$
$t_{4}^{xz}$	$-0.032\ (-0.034)$
$\epsilon ^{x}$	$0.870\ (0.776)$
$\epsilon ^{z}$	$0.351\ (0.409)$
$t_{4}^{x}$	$-0.064$
$t_{4}^{z}$	$-0.014$
$t_{5}^{x}$	$-0.015$
$t_{5}^{z}$	$-0.003$
$t_{5}^{xz}$	0.026
$t_{3}^{x}$	$-0.001$
$t_{3}^{z}$	0.033

We now compare the Fermi surfaces obtained from DFT+$U$ and ARPES experiments in both undoped ($n=1.5$) and hole-doped ($n=1.3$, as estimated in the ARPES experiments [40]) scenarios. Significant discrepancies between the DFT+$U$ results and ARPES data are apparent. For instance, the distance $d$ (marked by the red arrow in Fig. 2c) between two Fermi surface branches of the $\beta$ band near the $(\pi ,0)$ point is much smaller in ARPES than that in the DFT results. The $\gamma$ pocket around $(\pi ,\pi )$ seen in ARPES is much smaller than that in DFT+$U$, and its diffuse nature may indicate that the $d_{z^2}$ states are much less coherent than the $d_{x^2-y^2}$ states. We demonstrate that these discrepancies arise from the incomplete treatment of electron correlations in the DFT+$U$ approach.

The second part is the rotation-invariant Kanamori-type multi-orbital on-site interaction. The realistic intra-orbital Coulomb interaction $U_\mathrm{RPA}\approx 3.77$ eV and Hund’s coupling $J_{\mathrm{RPA}}\approx 0.56$ eV (see Table 3) are obtained according to the constrained random phase approximation (cRPA) [42], which counts in the screening effect from the rest energy bands out of the low-energy $e_g$ subspace. As the non-local interaction $V$ can screen $U$ further [20] while $J$ is unscreened, we tune $U$ close to $U_\mathrm{RPA}$ with fixed $J=J_{\mathrm{cRPA}}= 0.56$ eV to mimic this screening effect and also systematically investigate the effect of $U$ on the correlated electronic structure. By comparing the electronic structure calculated from CDMFT with that of the ARPES experiment, we find that the best fitting happens at $U \sim 3.6$ eV (shown below). In this regime, CDMFT shows clear quasi-particle (QP) bands, but with some small QP weight ($Z_{d_z^2} \sim 0.2 \ll 1$), which suggests that the system is away from a doped Mott insulator, but still has a significant correlation effect. In other words, our CDMFT results indicate that the thin film is an intermediate correlated system.

**Table 3.** Interaction parameters. The static intra-layer on-site Coulomb repulsion ($U$) and inter-layer non-local Column interaction $V$ (in units of electronvolts) calculated from cRPA. Here $\overline{U}$ is the orbital averaged value.


$U^x$	4.03
$U^z$	3.51
$\overline{U}$	3.77
$J$	0.56
$V_{\perp }^x$	1.04
$V_{\perp }^z$	1.31
$V_{\perp }^{x,z}$	1.13

### CDMFT studies

**Figure 3.**
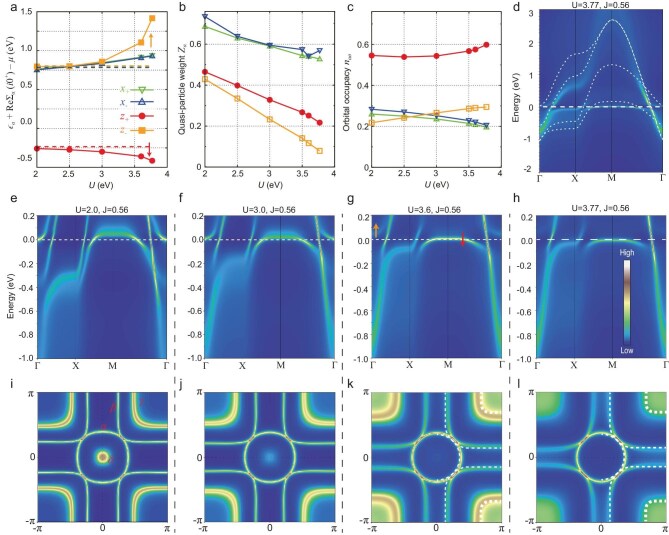
The $U$ dependence of the CDMFT results at temperature $T=200$ K, filling $n=1.3$ per Ni (in $e_g$ orbitals) and Hund coupling $J=0.56$ eV. (a) The effective energy level $\epsilon ^{\text{eff}}_{\alpha } = \epsilon _{\alpha }+\mathrm{Re}\Sigma_\alpha{(i0^+)} - \mu$ for each bonding and anti-bonding orbital as a function of $U$ ($\mu$ is the chemical potential). The dashed lines mark the bare energy levels $\epsilon _{\alpha }$ from DFT. (b) The quasi-particle spectral weight $Z_{\alpha }$ (degenerate in spin). (c) The orbital occupancy per spin $n_{\alpha \sigma }$. (d–h) The momentum-resolved spectral function $A({\bf k},\omega )$ at indicated $U$. We show $A({\bf k},\omega )$ in the large energy window $-2<\omega <3$ for $U=3.77$ eV in (d) and in the enlarged energy window $-1<\omega <0.2$ for $U=2.0$, 3.0, 3.6 and 3.77 eV in (e–h), respectively. The thin white dashed line in (d) indicates the DFT band of Ni-$e_g$ orbitals. The thick dashed line in (d–h) marks the Fermi energy. (i–l) The corresponding Fermi surface $A({\bf k},0)$ at indicated $U$ shown in (e–h). In panels (k–l), the extracted ARPES Fermi surfaces [37] are overlaid as the white dashed line. Only the right half is shown for clarity. The energy unit is in electronvolts (eV).

The main results of CDMFT are summarized in Fig. 3, which in detail demonstrates how the physical quantities like quasi-particle weight, occupancy, effective energy level, spectral function and Fermi surfaces vary as one increases $U$. The calculations are performed at $T=200$ K and $n=1.3$ per site, which is close to the filling 1.28 estimated in the ARPES experiment [40]. More results on doping and temperature dependence can be found in Section S3, especially the results at lower temperatures like $T= 50$ K, which only have quantitative differences from the results at $T= 200$ K.

As one increases $U$, we observe an increase in the effective energy splitting $\epsilon ^{\text{eff}}_{z_-}-\epsilon ^{\text{eff}}_{z_+}$ between the $z_+$ and $z_-$ orbitals, where $\epsilon ^{\text{eff}}_{\alpha } = \epsilon _{\alpha }+\mathrm{Re}\Sigma_\alpha {(i0^+)} - \mu$. Thus, the effective energy level $\epsilon ^{\text{eff}}_{z_-}$ ($\epsilon ^{\text{eff}}_{z_+}$) of the $z_-$ ($z_+$ ) orbital is pushed upward (downward), as seen in Fig. 3a. For $U\le$ 3.0 eV, $\epsilon ^{\text{eff}}_{z_\pm }$ deviates slowly from its bare value $\epsilon _{z_\pm }$ (dashed lines). The deviation increases rapidly when $U\gtrsim 3.6$ eV, as indicated by the arrows. This is consistent with the picture of strong inter-layer electronic correlations between the $z$ orbitals [28], which becomes more prominent as $U$ increases. On the contrary, $\epsilon ^{\text{eff}}$ for the $x$ orbitals remains gradually increasing even for larger $U$. The on-site Coulomb repulsion $U=3.6$ eV could be regarded as a characteristic interaction strength where the correlation effect becomes significant.

The $U$-dependent orbital occupancy per spin is given in Fig. 3c. There is a self-doping effect with the electrons transferring from the $x$ orbitals to the $z$ orbitals (note that $n_d=1.3$ fixed). As $U$ is tuned from 2 to 3.77 eV, the total filling in the $z$ orbitals increases from $\sim\!\! 0.76$ to $\sim\!\! 0.9$, while the total filling in the $x$ orbitals decreases from $\sim\!\! 0.54$ to $\sim\!\! 0.4$. The quasi-particle weight $Z$ of the $x_{\pm }$ orbitals ranges from $\sim\!\! 0.7$ to $\sim\!\! 0.5$ as $U$ is tuned down, which results from a small filling per spin ($\sim\!\! 0.3$ to $\sim\!\! 0.2$). The $Z$ of $z_\pm$ orbitals decreases from $\sim\!\! 0.45$ to $\sim\!\! 0.2$ for the $z_+$ orbital and to $\sim\!\! 0.08$ for the $z_-$ orbital, showing that $z$ orbitals are much more correlated than $x$ orbitals. The reason for this is that the self-doping effect from the $x$ to $z$ orbital makes the filling in each $z$ orbital approach a half filling per spin (from $\sim\!\! 0.38$ at $U=2$ to $\sim\!\! 0.45$ at $U=3.77$). Interestingly, as one increases $U$, $n_{z_-\sigma }$ becomes even larger even though $\epsilon ^{\text{eff}}_{z_-}$ is shifted to a higher energy, resulting from the formation of Hubbard bands (see Section S10).

Associated with the enhanced effective level splitting as $U$ increases, the ${\bf k}$ spectral functions $A({\bf k},\omega )$ and Fermi surfaces evolve correspondingly. From Fig. 3e–h, one can see that both the $\delta$ band at the $\Gamma$ point and the $\gamma$ band at the $M$ point at low energy become more flat and gradually fade away (being less coherent) from the Fermi energy. First, as mentioned before, there is a downward or upward shift of $\epsilon ^{\text{eff}}_{z_\pm }$. Second, the quasi-particle spectral weight (see Fig. 3b) decreases, which especially makes the $\gamma$ band more flat near the Fermi energy. Furthermore, the scattering rate is enhanced when $U$ increases (not shown). As a result, the Fermi surface of the $\delta$ electron pocket gradually disappears, as seen from Fig. 3i–l. Simultaneously, the $\gamma$ hole pocket also shrinks in size and becomes less coherent (as evidenced by the decreased intensity of the colormap). At $U=3.77$, the $\gamma$ band becomes flat near the $M$ point, and its band top touches the Fermi energy, which gives rise to the Fermi surface shown in Fig. 3l. The $A({\bf k},\omega )$ and Fermi surfaces at $U=3.6$ to $U=3.77$ eV shown in Fig. 3g, k quantitatively match all the key features seen in ARPES Fermi surfaces, as indicated by the white dashed lines in Fig. 3k. (Doping and temperature dependence of the physical quantities in CDMFT are presented in Section S3.) The agreement with ARPES data originates from the enhanced effective level splitting for $U\gtrsim 3.6$ eV, suggesting that the thin film is in an intermediate coupling regime.

### Paring mechanism

The superconducting pairing symmetry is studied with a modified RPA approach (see Section S6), where the quasi-particle Hamiltonian from the CDMFT is taken as an input (see the Methods section below and Section S4 for details). We assume that the effective interaction used in RPA is in the same form as that in Equation (3) below and we fix ${J_{\textrm {eff}}} = U_{\textrm {eff}}/6$ in the calculations. The effective interaction strength ${U_{\textrm {eff}}}$ is a tuning parameter. It could be regarded as the strength of some kinds of residual interaction renormalized by a factor $Z^2$, where $Z$ is the quasi-particle weight. Since $Z$ of the $d_{z^2}$ orbital is quite small ($\sim\!\! 0.18$ for $U = 3.6$ eV), the effective interaction strength ${U_{\textrm {eff}}}$ should also be very small.

**Figure 4.**
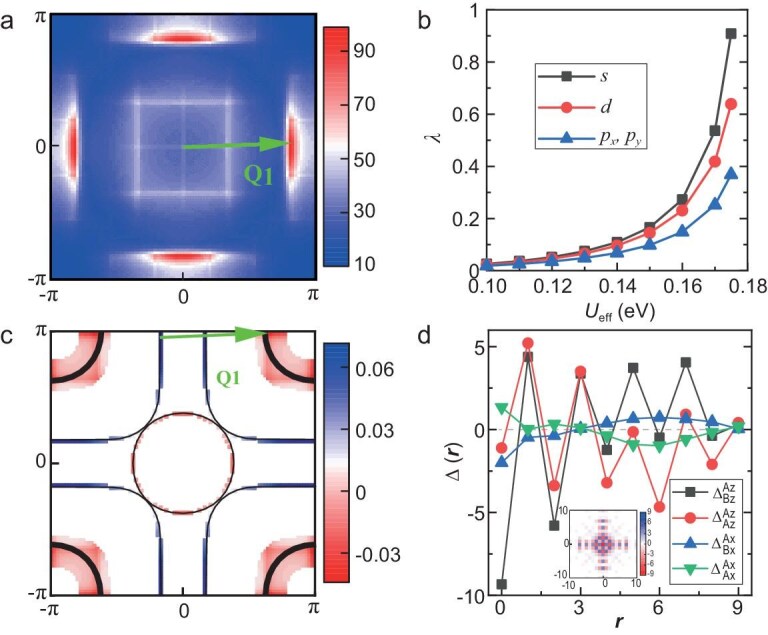
The CDMFT+RPA calculation results on a ${96\times 96}$ square lattice at fixed temperature ${T=0.001}$ eV (∼11.6 K), filling ${n=1.3}$ per Ni-${e_g}$ and bare interaction strength ${U=3.6}$ eV. (a) The distribution of the largest eigenvalue $\chi ^{(s)}({\bf q})$ of the RPA-renormalized spin susceptibility matrix in the Brillouin zone for ${U_{{\rm eff}}}=0.17$ eV. Here ${\bf Q}_{1}$ is the representative wave vector of eight equivalent distribution peaks related by the $D_{4h}$ point group. (b) The largest eigenvalue $\lambda$ of the effective pairing interaction vertex matrix as a function of the effective interaction strength $U_{{\rm eff}}$ for leading $s$, $d$ and degenerate $p_{x}$ and $p_{y}$ wave pairings. (c) The distribution of the leading $s^{\pm }$-wave pairing gap function near the Fermi surface for ${U_{{\rm eff}}}=0.17$ eV. The black thin lines denote the Fermi surface. The wave vector ${\bf Q}_{1}$ in (a) and the Fermi surface nesting vector in (c) are identical. (d) The real space $\mathbf {r}$ dependence of the $s^{\pm }$-wave pairing gap function in the orbital basis for ${U_{{\rm eff}}}=0.17$ eV. Here $\mathbf {r}$, the distance in units of the lattice, is constant between the two orbitals from two unit cells involved in the pairing. The inset shows the spatial distribution of $\Delta _{Bz}^{Az}$. We denote by $\Delta _{\tau ^{\prime }\gamma ^{\prime }}^{\tau \gamma }$ the pairing between the $\gamma$ orbital on the $\tau$ layer and the $\gamma ^{\prime }$ orbital on the $\tau ^{\prime }$ layer. The other input parameters are all from CDMFT.

The RPA results for $U = 3.6$ eV and $n = 1.3$ are summarized in Fig. 4. In Fig. 4a, we show the typical RPA-renormalized spin susceptibility. The dominant wave vector ${\bf Q}_{1}$ corresponds to the nesting between the $\beta$ pocket and $\gamma$ pocket, as shown in Fig. 4c. In Fig. 4b, we show the dependence of the effective pairing strength [43] $\lambda$ on ${U_{\textrm {eff}}}$ for various pairing symmetries. It is clear that a larger ${U_{\textrm {eff}}}$ leads to a stronger superconducting instability. The leading pairing symmetry is always an $s^\pm$ wave, where the gap function on the Fermi surface is depicted in Fig. 4c. The coincidence of ${\bf Q}_{1}$ in Fig. 4a and the one that connects the largest gap in magnitude in Fig. 4c supports the spin-fluctuation-mediated pairing mechanism. In real space, the strongest pairing is between the inter-layer $d_{z^{2}}$ orbitals, as shown in Fig. 4d, which is consistent with the fact that the Fermi surface patches connected by ${\bf Q}_{1}$ on the $\beta$ and $\gamma$ bands shown in Fig. 4c are mainly formed by $d_{z^{2}}$ orbitals. We also perform similar calculations for $3\le U\le 3.77$, and $s^{\pm }$-wave pairing is always dominant (see Section S4 for more details). All in all, the most possible pairing symmetry is $s^{\pm }$-wave pairing for realistic parameters.

## DISCUSSION

First, we discuss the extent of the correlation effects in bilayer nickelate films. The on-site Coulomb repulsion from cRPA is $U \sim 3.77$ eV, consistent with the typical value of $U = 3$–4 eV for transition metals. Because of the strong inter-layer coupling, the multi-orbital tight-binding model involves four bands with a total bandwidth around 4 eV, which is comparable to the on-site Hubbard $U$. The situation is similar to that in the iron-based superconductor [44–47], suggesting that bilayer nickelate systems also lie in an intermediate correlation regime.

Although the correlation is moderate, its effects are non-negligible. The weak-coupling fluctuation-exchange (FLEX) approximation [32,48,49] at the same filling $n=1.3$ fails to reproduce ARPES Fermi surfaces. As a weak coupling approach, FLEX calculations are restricted to $U\le 1.8$ eV, beyond which the method may lose convergence. In the weak $U$ region, FLEX can reproduce the Fermi surface obtained in CDMFT for $U\le 3.0$ (see Section S5). However, FLEX fails to see a Fermi surface topology similar to Fig. 3k obtained in CDMFT at $U=3.6$ eV. This indicates that FLEX cannot describe the strong inter-layer correlation, which plays a crucial role in the electronic structure of bilayer nickelate thin films.

Next, we discuss the comparison between strained thin films and pressurized bulk. The coupling between inter-layer $d_{z^2}$ orbitals ($t_\perp ^z/J_\perp ^z$) is believed to be crucial in pressurized bulks [16,22,32]. However, thin films have a much longer inter-layer distance than that in pressurized bulks, which leads to much weaker inter-layer couplings, say about $30\%$ lower for $t^z_{\perp }$ and $50\%$ lower for $J^z_{\perp }$ (since it is proportion to $(t^z_{\perp })^2$). It would be a challenge for the theories strongly depending on $t^z_{\perp }$ and/or $J^z_{\perp }$ to explain the same order-of-magnitude onset ${T_\mathrm{C}}$ of the two systems. In contrast, although DFT calculations suggest a distinct Fermi surface topology for the pressurized samples, the CDMFT-derived Fermi surface (which incorporates correlation effects and aligns with ARPES data) resembles that of the high-pressure case at the same filling $n=1.3$ (see Section S8). Therefore, it would be much easier to understand why thin films have the same order-of-magnitude onset ${T_\mathrm{C}}$ with a much smaller $t^z_{\perp }$ given that the correlated effect has been included correctly.

Finally, we would like to emphasize the quantitative effectiveness of the DFT+cRPA+CDMFT approach in the study of unconventional superconductors with weak to intermediate correlation effects. Simply with lattice parameter inputs from experiments, the *ab initio* calculations without parameter fine-tuning match ARPES results quantitatively. The following RPA or similar techniques may provide a qualitatively accurate description of the pairing symmetry on top of the precise correlated electronic structures. We believe that this approach can be applied to a system with intermediate correlation and far from a doped Mott insulator, where quasi-particles and the Fermi surface can still be well defined. In this way, we can derive an effective quasi-particle Hamiltonian from DFT+cRPA+CDMFT, based on which we can discuss pairing in RPA. Therefore, this approach is feasible for studying superconductivity in weakly and moderately correlated systems within first-principles calculations. Its wide applicability in various materials may shift the paradigm of searching for unconventional superconductors.

## METHODS

### STEM experiment

All electron microscopy analyses were conducted using an FEI Themis Z transmission electron microscope operated at 200 kV. This microscope is equipped with a Cs probe corrector (DCOR) and a high-brightness field-emission gun with a monochromator to enhance resolution and contrast. For STEM, high-angle annular dark field (HAADF) and annular bright field (ABF) imaging, the inner and outer acquisition angles ($\beta$1 and $\beta$2) were set to 90 and 200 mrad for HAADF images, and 12 and 23 mrad for ABF images, respectively. The convergence angle was maintained at 25 mrad. The beam current was adjusted to approximately 40 pA for HAADF and ABF imaging. Cross-sectional STEM specimens were prepared using an FEI Helios G4 HX dual-beam focused-ion-beam/scanning electron microscope system. The simulation of ABF images was performed using the multi-slice method implemented in the QSTEM software. The simulation parameters were carefully set to match those used during the electron microscopy characterization, ensuring that the simulated images accurately reflect the experimental conditions.

### *Ab initio* multi-orbital Hubbard model

We construct the realistic multi-orbital Hubbard model for the half-UC thin film, including the non-interacting TB model and the on-site interaction term:


(1) \begin{equation*}
H=\sum _{{\bf k \sigma }}\Psi _{{\bf k \sigma }}^{\dagger }H^{0}({\bf k})\Psi _{\bf k \sigma }+\sum _{i\tau }H_{i\tau }^{\mathrm{int}}.
\end{equation*}

We use $i$ to enumerate the unit cells, $\tau$ the layer indices ($A$ for the bottom layer, $B$ for the top layer), $\gamma$ the orbital indices ($x$ for $d_{x^2-y^2}$ orbital and $z$ for $d_{z^2}$ orbital) and $\sigma$ the spin states $\uparrow$ and $\downarrow$. Here $\Psi _{\bf k \sigma }=(d_{\bf k Ax\sigma },d_{\bf k Az\sigma },d_{\bf k Bx\sigma },d_{\bf k Bz\sigma })^{T}$.

The low-energy bands’ TB model is obtained from the maximally localized Wannier functions constructed with the wannier90 package [50,51]. The TB model Hamiltonian in ${\bf k}$ space has the same matrix structure as in [14]:


(2a) \begin{eqnarray*}
H^0({\bf k}) =
\left({\begin{array}{cc}
H^0_{A}({\bf k}) &\quad H^0_{AB}({\bf k})\\
H^0_{AB}({\bf k}) &\quad H^0_{A}({\bf k}) \end{array}}\right),
\end{eqnarray*}

(2b) \begin{eqnarray*}
H^0_{A}({\bf k})=
\left({\begin{array}{cc}
T_{{\bf k}}^{x} &\quad V_{{\bf k}}\\
V_{{\bf k}} &\quad T_{{\bf k}}^{z} \end{array}}\right),
\end{eqnarray*}

(2c) \begin{eqnarray*}
H^0_{AB}({\bf k})=
\left({\begin{array}{cc}
T_{{\bf k}}^{\prime x} &\quad V_{{\bf k}}^{\prime }\\
V_{{\bf k}}^{\prime } &\quad T_{{\bf k}}^{\prime z} \end{array}}\right),
\end{eqnarray*}

with


\begin{eqnarray*}
T_{{\bf k}}^{x/z} &=& 2t_{1}^{x/z}(\cos k_{x}+\cos k_{y}) \\
&&\quad +\,2t_{4}^{x/z}(\cos 2k_{x}+\cos 2k_{y}) \\
&&\quad +\,2t_{5}^{x/z}(\cos 3k_{x}+\cos 3k_{y}) \\
&&\quad +\,4t_{2}^{x/z}\cos k_{x}\cos k_{y}+\epsilon _{x/z}, \\
V_{{\bf k}} &=& 2t_{3}^{xz}(\cos k_{x}-\cos k_{y}) \\
&&\quad +\,2t_{5}^{xz}(\cos 2k_{x}-\cos 2k_{y}), \\
T_{{\bf k}}^{\prime x/z} &=& t_{\perp }^{x/z}+2t_{3}^{x/z}(\cos k_{x}+\cos k_{y}), \\
V_{{\bf k}}^{\prime } &=& 2t_{4}^{xz}(\cos k_{x}-\cos k_{y}).
\end{eqnarray*}

Here, $T^{x/z}_{\bf k}$ ($T^{\prime x/z}_{\bf k}$) is the intra-layer (inter-layer) intra-orbital hopping, and $V_{{\bf k}}$ ($V_{{\bf k}}^{\prime }$) is the intra-layer (inter-layer) inter-orbital hopping. The hopping parameters are summarized in Table 2.

The interacting part of Equation (1) reads $H^{\mathrm{int}} =\sum _{i\tau } H^{\mathrm{int}}_{i\tau }$ with $H^{\rm int}_{i\tau }$ the Kanamori-type two-orbital on-site interaction:


(3) \begin{eqnarray*}
H_{\mathrm{i\tau }}^{\mathrm{int}} &=& \sum _{\gamma }U_{\gamma } n_{i\tau \gamma \uparrow } n_{i\tau \gamma \downarrow }\nonumber \\
&& +\, \sum _{\gamma <\gamma ^{\prime },\sigma \sigma ^{\prime }}(U^{\prime }-\delta _{\sigma \sigma ^{\prime }}J) n_{i\tau \gamma \sigma }n_{i\tau \gamma ^{\prime }\sigma ^{\prime }}\nonumber \\
&& -\, J\sum _{\gamma <\gamma ^{\prime }}(c_{i\gamma \downarrow }^{\dagger }c_{i\gamma ^{\prime }\uparrow }^{\dagger } c_{i\gamma ^{\prime }\downarrow }c_{i\gamma \uparrow }+\mathrm{H.c.})\nonumber \\
&& -\,J\sum _{\gamma <\gamma ^{\prime }}(c_{i\gamma ^{\prime }\uparrow }^{\dagger }c_{i\gamma ^{\prime }\downarrow }^{\dagger } c_{i\gamma \uparrow }c_{i\gamma \downarrow }+\mathrm{H.c.}).
\end{eqnarray*}

The on-site interaction $H^{\rm int}_{i\tau }$ contains the intra-orbital ($U$), inter-orbital ($U^\prime =U-2J$) Coulomb repulsions, and the Hund coupling $J$. The second line in Equation (3) shows the spin-flip and pair-hopping terms. Realistic values of $U$ and $J$ are obtained by cRPA [42], which accounts for the screening effect from the rest energy bands out of the low-energy $e_g$ subspace. The results obtained from cRPA are listed in Table 3. Although there is slight anisotropy between $U_x$ and $U_z$, we take their average $\overline{U}=\sum _\gamma U_\gamma / 2 \approx 3.77$ eV for both the orbital and Hund coupling $J$ as $J_{\mathrm{cRPA}} = 0.56$ eV, which is slightly different from those of the high-pressure bulk crystal (3.79 and 0.61 eV) [20]. We also listed the inter-layer Coulomb repulsion $V$ in the range $\sim\!\! 1.0$–1.3 eV in the thin film, roughly 30% of $\overline{U}_{\mathrm{cRPA}}$. The crystal structure TB model obtained from wannier90 with all long-range hopping integrals and the cRPA interaction tensor $U_{ijkl}$ are freely available from Zenodo [52].

### CDMFT calculation

We apply the CDMFT [53,54] method to study the correlated electronic structure of the half-UC thin film. In the bonding (${z_+}$, ${x_+}$) and anti-bonding (${z_-}$, ${x_-}$) bases, the cluster impurity problem is equivalent to an effective four-orbital single impurity model with diagonal crystal field splitting among these orbitals. The numerically exact hybridization-expansion continuous-time quantum Monte Carlo algorithm [55,56] is adopted to solve the quantum impurity model. To calculate the momentum-resolved spectral function, we adopt the maximum-entropy analytic continuation method to continue the self-energy from the Matsubara frequency axis to the real frequency axis [57].

### CDMFT+RPA calculation

The CDMFT+RPA method is a modified RPA approach in which the infinite sum of bubble diagrams, i.e. the RPA, is performed upon the quasi-particle Hamiltonian from CDMFT. We assume that the effective interaction has the same form as the bare interaction, with strength only renormalized by the quasi-particle weight. The advantage of the CDMFT+RPA method is that a small effective interaction actually corresponds to an intermediate bare interaction. The RPA-renormalized spin and charge susceptibilities can investigate spin-density-wave, charge-density-wave and pairing instabilities. There exists a critical interaction strength above which RPA results become unreliable. Near critical values, enhanced spin fluctuations can mediate an effective attraction between quasi-particles close to the Fermi surface and serve as glue for forming Cooper pairs. The effective attraction is treated by mean-field theory to derive the self-consistent superconducting (SC) gap equation. Near the SC critical temperature $T_{\rm c}$, the gap equation can be linearized and becomes a standard eigenvalue problem. The largest eigenvalue of the effective pairing interaction vertex matrix determines $T_{\rm c}$, and corresponding eigenvectors give the distribution of gap functions, from which we can determine the pairing symmetry [43].

## NOTE ADDED

After the submission of our manuscript to arXiv (https://arxiv.org/abs/2501.06875), we became aware of three independent studies [58–60] appearing after our work.

Shao *et al.* [58] performed a DFT+RPA calculation, Le *et al.* [59] studied the electronic structures and the instabilities of a 3-UC thin film on the substrate based on DFT and functional renormalization group calculations, while Shi *et al.* [60] systematically examined both carrier doping and film thickness in thin films based on DFT+$U$ calculations. In comparison to their studies, our results are based on more accurate lattice structures measured with STEM experiments and include the very important correlated electronic structure obtained with CDMFT, and we also reproduce the Fermi surface by ARPES experiment.

## Supplementary Material

nwaf253_Supplemental_File
