# Supplementary material for: Correlated electronic structures and unconventional superconductivity in bilayer nickelate heterostructures
Source: Natl Sci Rev. 2025 Jun 23;12(10):nwaf253. doi: 10.1093/nsr/nwaf253 (PMC12485619; doi:10.1093/nsr/nwaf253)
Supplement: nwaf253_Supplemental_File [file nwaf253_supplemental_file.pdf]

Supplementary Information for
Correlated electronic structures and unconventional superconductivity in bilayer nickelate heterostructures

Changming Yue[1,2,3,*#], Jian-Jian Miao[1,2,#], Haoliang Huang[1,2,#], Yichen Hua[1,#], Peng Li[1,2], Yueying Li[1], Guangdi Zhou[1], Wei Lv[1], Qishuo Yang[1], Fan Yang[4], Hongyi Sun[2], Yu-Jie Sun[1,2], Junhao Lin[1,2], Qi-Kun Xue[1,2,5], Zhuoyu Chen[1,2,*], Wei-Qiang Chen[1,2,*]

[1]*State Key Laboratory of Quantum Functional Materials, Department of Physics, and Guangdong Basic Research Center of Excellence for Quantum Science, Southern University of Science and Technology, Shenzhen 518055, China*
[2]*Quantum Science Center of Guangdong-Hong Kong-Macao Greater Bay Area, Shenzhen 518045, China*
[3]*Guangdong Provincial Key Laboratory of Advanced Thermoelectric Materials and Device Physics, Southern University of Science and Technology, Shenzhen, 518055, China*
[4]*School of Physics, Beijing Institute of Technology, Beijing 100081, China*
[5]*Department of Physics, Tsinghua University, Beijing 100084, China*

#These authors contribute equally.
*Corresponding authors: yuecm@sustech.edu.cn, chenzhuoyu@sustech.edu.cn, chenwq@sustech.edu.cn

SM1. Additional structural information

The lattice structure parameters were taken from a superconducting $La_{2.85}Pr_{0.15}Ni_2O_7$ film and a non-superconducting $La_3Ni_2O_7$ film grown on $SrLaAlO_4$ substrates[1]. Fig. S1 shows the annular bright-field (ABF) images of the cross-section of $La_{2.85}Pr_{0.15}Ni_2O_7$ sample, projected along $[100]_{pc}$, $[010]_{pc}$ and $[110]_{pc}$. We analyzed the positions of oxygen atoms in these three projections and selected part of the data to show in Fig. 1, which reflects that the $La_{2.85}Pr_{0.15}Ni_2O_7$ thin film has almost no oxygen octahedral rotation under compressive epitaxial strain. The atomic spacing used for modeling is obtained by measuring directly on High-angle Annular Dark Field (HAADF) images with calibration from XRD lattice constants. Fig. S2 identifies several key atomic spacing values of $La_{2.85}Pr_{0.15}Ni_2O_7$ and $La_3Ni_2O_7$ films, which are also listed in Tables 1 and Tables S1. At the same time, considering the error of the electron microscope in the measurement, the listed values show only two significant digits after the decimal point.

SM2. Ab initio Calculations

We build the crystal structure of the half-UC ultra-thin film of $La_3Ni_2O_7$ according to the structural parameters (see Table 1 in the main text) of the ultra-thin film of $La_{2.85}Pr_{0.15}Ni_2O_7$. To be specific, we choose the lattice constant $a = b = 3.7544$Å, inter-layer La-La distance 3.705Å, inter-layer Ni-Ni distance 4.28Å. The middle La layer is placed in the middle of the Ni-bilayer. Furthermore, the inter-layer Ni-O-Ni and La-O-La angles are 180° as the ultra-thin film has C_4 rotation symmetry. As for the c axis, we set $|c|$=40 Å to make the length of the vacuum $\sim$ 30Å long enough to simulate thin film in the periodic structure. The positions of oxygen are placed according to its high-pressure structure. The *ab initio* density-function theory (DFT) calculations, including structure relaxation and band structure, are performed using the VASP package[2–4], in which we use the Perdew-Burke-Ernzerhof (PBE) exchange-correlation functional[5]. As the ions are heavier in mass and the strain from the substrate confines their positions, we relax only the oxygen atoms in the built structure while keeping the La and Ni atoms fixed. In the DFT calculations, we set the energy cutoff for the plane-wave basis 550 eV, k-mesh grid 11×11. The atomic positions of oxygen are relaxed until the norms of all the forces are smaller than 0.001 eV/Å.

We adopt the virtual crystal approximation (VCA) to simulate the Sr-doping effect. As for where to dope Sr, there are several different methods. Here, we tried two different kinds of doping: doping Sr only to the middle La layer and doping Sr uniformly to all La layers. In the main text, the band structure and tight-binding model correspond to the results of doping Sr only to the middle La layer. The following shows that the low-energy bands are not sensitive to these two doping methods. First, we confirm that the low-energy bands of Ni-e_g orbitals are nearly rigid, especially the bands near Fermi energy, as shown by Fig. S3a. Second, we confirm that the low-energy bands Ni-e_g orbitals are insensitive to how we dope Sr to La when the average hole doping level per Ni-layer is the same, as shown by Fig. S3b (doping Sr only to the middle La layer) and (c) (doping Sr uniformly to all La layer). We also directly compare the DFT+U bands between two different methods of Sr-doping in Fig. S3d, confirming that the low-energy bands of Ni-e_g orbitals are almost identical even though the high energy bands are different. The relaxed atomic positions corresponding to doping Sr only in the middle La layer are tabulated in Table S2. In both Sr-doping methods, the low-energy bands are insensitive to U as shown in Fig. S3b-c. Here, the effect of U is to push downward the already full-filled t_{2g} bands.

As the low-energy Ni-e_g bands are almost identical to each other, no matter where Sr is doped and whether or not U is added, these TB parameters calculated with DFT+U can also be safely regarded as that of the DFT bands, as usually used in the literature. The directions of all hopping integrals are schematically demonstrated in Fig.S4. Besides two key differences between the TB model of the half-UC ultra-thin film and that of high-pressure bulk, we introduce six additional long-range hopping parameters. For example, we have the inter-layer next nearest neighbor (NNN) hopping between the x-orbitals t_3^x=0.0332 eV, the intra-layer NNN hopping between x-orbitals t_4^x=-0.0639 eV. There is also inter-layer inter-orbital NNNN hopping t_5^{xz}=0.0255 eV.

SM3. Doping and Temperature Dependence of CDMFT results

Fig. S5 shows the doping dependence of $A(\mathbf{k},\omega)$ and FS at $U = 3.6$ eV and indicated fillings. As one decreases the filling $n = 1.4$ to $n = 1.2$ (equivalent to increasing hole doping from 0.1 hole to 0.3 hole per Ni-e_g orbitals), one can see that the γ (δ) band moves upward (downward), which can be explained by a reduced effective level splitting between z_+ and z_- orbitals. The Fermi surface at $n = 1.2$ (Fig. S5f resembles the one seen in DFT (Fig. 2c) at $n = 1.33$. The reason is that the inter-orbital correlation strength decreases when the filling per orbital is reduced. As a result, the γ hole (δ electron) pocket gradually appears and expands as n reduces.

Fig. S6 shows the temperature dependence of $A(\mathbf{k},\omega)$ and FS at $U = 3.6$, which changes only qualitatively as one decreases of temperature from T=200 K to T=50 K. The main difference is that the low-energy electrons become more coherent at lower T, leading to sharper low-energy bands and sharper Fermi surfaces.

SM4. CDMFT+RPA

CDMFT+RPA is based on the quasi-particle Hamiltonian from CDMFT. Our CDMFT calculation shows that the Fermi surface topology can be greatly affected at larger U>3 eV. As the low-energy excitations mainly control the pairing, a better starting point for RPA is to use the quasi-particle Hamiltonian obtained from CDMFT. We call such treatment CDMFT+RPA. The quasi-particle Hamiltonian $\tilde{H}^{\mathrm{QP}}_{\mathbf{k}}$ is obtained from the quasi-particle approximation of self-energy

$$\tilde{\mathbf{\Sigma}}^{\mathrm{QP}}(\omega) \approx \mathrm{Re}\tilde{\mathbf{\Sigma}}(i0^+) + (\mathbf{I}_4 - \tilde{\mathbf{Z}}_4^{-1})(\omega + i\eta) \tag{1}$$

Here, we do not distinguish spins as they are degenerate (dimension 4 for each spin). ˜ means all the quantities are in the bonding and anti-bonding basis. In this basis, the zero-frequency self-energy $\tilde{\mathbf{\Sigma}}(i0^+)$ and quasi-particle spectral weight $\tilde{\mathbf{Z}}$ obtained from CDMFT are both diagonal. The quasi-particle Green's function reads

$$\tilde{\mathbf{G}}^{\mathrm{QP}}(\mathbf{k},\omega) = \Big[(\omega + i\eta + \mu)\mathbf{I}_4 - \tilde{H}_{\mathbf{k}} - \tilde{\mathbf{\Sigma}}^{\mathrm{QP}}(\omega)\Big]^{-1} \tag{2}$$

The corresponding quasi-particle Hamiltonian is

$$\tilde{H}^{\mathrm{QP}}_{\mathbf{k}} = \tilde{\mathbf{Z}}^{1/2}[\tilde{H}_{\mathbf{k}} - \mu\mathbf{I}_4 + \mathrm{Re}\tilde{\mathbf{\Sigma}}(i0^+)]\tilde{\mathbf{Z}}^{1/2} \tag{3}$$

FIG. S7 illustrates that the correspondence between $\mathbf{Q}_1$ in the distribution of RPA-renormalized spin susceptibility and FS nesting still holds for different parameters. As the shape of FS changes for different fillings and bare interactions in CDMFT, the wave vector $\mathbf{Q}_1$ also varies accordingly. FIG. S7b,d,f,h demonstrate that the gap functions on FS patches connected by $\mathbf{Q}_1$ possess opposite signs, which is similiar to the $d_{x^2-y^2}$-wave pairing in the cuprates and the $s^{\pm}$-wave pairing in the iron-based superconductors.

FIG. S8 presents the dependence of λ on U_{eff} for $U = 3.77$ eV, $U = 3.6$ eV, and $U = 3$ eV. The leading pairing symmetry is always the $s^{\pm}$ wave pairing before the RPA-renormalized spin susceptibility diverges. For small bare interaction in FIG. S8c, there is obvious competition between $s^{\pm}$-wave and d_{xy}-wave pairings. With stronger bare interaction, only the $s^{\pm}$-wave pairing instability is leading as shown in FIG. S8a. Both results further support the main conclusion that the most possible pairing symmetry is $s^{\pm}$-wave pairing for realistic parameters in the main text.

SM5. FS in Fluctuation-exchange approximation

Fluctuation-exchange (FLEX) approximation[6] has been developed to study the pairing instability of Hubbard models. FLEX is, by nature, a weak-coupling approach, and it breaks down when U is stronger. Compared with normal RPA, FLEX can reach slightly higher correlation strength as the self-energy is incorporated. Here, we apply the FLEX calculation to the same multi-orbital Hubbard model of the ultra-thin film using a modified FLEX package developed by Witt *et al.*[7]. The Fermi surfaces at filling $n = 1.3$ are shown in Fig. S9a-b. It turns out the δ-pocket remains at Γ point when U is increased from $U = 1$ to $U = 1.8$ above which FLEX breaks down. While the FS of FLEX at the same filling $n = 1.3$ is similar to that given by CDMFT at $U \leq 3$ (Fig. 3i-j), FLEX fails to reproduce FS as in Fig. 3l in the safe radius of U. Note that the largest U that FLEX can reach depends on the filling and density of states. We confirm that our FLEX calculation can reach $U \leq 3$ for the TB model of high-pressure bulk crystal, which has a less flat γ-band than the ultra-thin film.

SM6. Multi-orbital RPA formalism

The Hamiltonian of the multi-orbital Hubbard model contains noninteracting and interacting parts

$$H = H_0 + H_{\text{int}}. \tag{4}$$

For the noninteracting case ($U = U' = J = 0$), define the bare susceptibility as follows

$$\chi^{(0)pq}_{st}(\vec{q},\tau) \equiv \frac{1}{N}\sum_{\vec{k}_1\vec{k}_2}\left\langle T_\tau c_p^\dagger(\vec{k}_1,\tau)c_q(\vec{k}_1+\vec{q},\tau)c_s^\dagger(\vec{k}_2+\vec{q},0)c_t(\vec{k}_2,0)\right\rangle_0 \tag{5}$$

where $p,q,s,t = 1,\ldots,4$ are combined orbital-layer indices. Here, 1 and 2 represent the two orbits of the top layer, 3 and 4 represent the two orbits of the bottom layer, T_τ is the imaginary-time-ordered product. After the Fourier transform to the imaginary frequency space, the bare susceptibility $\chi^{(0)}$ can be expressed as

$$\chi^{(0)pq}_{st}(\vec{q},i\omega_n) = \frac{1}{N}\sum_{\vec{k}}\sum_{\alpha\beta}\xi_p^{\alpha,*}(\vec{k})\xi_q^{\beta}(\vec{k}+\vec{q})\xi_s^{\beta,*}(\vec{k}+\vec{q})\xi_t^{\alpha}(\vec{k}) \times \frac{n_F(\epsilon^{\beta}_{\vec{k}+\vec{q}}) - n_F(\epsilon^{\alpha}_{\vec{k}})}{i\omega_n + \epsilon^{\alpha}_{\vec{k}} - \epsilon^{\beta}_{\vec{k}+\vec{q}}}, \tag{6}$$

where $\alpha, \beta = 1, \ldots, 4$ are band indices, $\epsilon^{\alpha}_{\vec{k}}$ and $\xi^{\alpha}(\vec{k})$ are the α-th eigenvalue and eigenvector of the noninteracting Hamiltonian matrix, respectively, and n_F is the Fermi-Dirac distribution function. For the interacting case, define the $(\chi^{(s)})$ and charge $(\chi^{(c)})$ susceptibilities as follows

$$\chi^{(c)pq}_{st}(\vec{q}, \tau) \equiv \frac{1}{2N} \sum_{\vec{k_1}\vec{k_2}\sigma\sigma'} \left\langle T_\tau c^{\dagger}_{p\sigma}(\vec{k_1}, \tau) c_{q\sigma}(\vec{k_1} + \vec{q}, \tau) c^{\dagger}_{s\sigma'}(\vec{k_2} + \vec{q}, 0) c_{t\sigma'}(\vec{k_2}, 0) \right\rangle, \tag{7}$$

$$\chi^{(s^z)pq}_{st}(\vec{q}, \tau) \equiv \frac{1}{2N} \sum_{\vec{k_1}\vec{k_2}\sigma\sigma'} \sigma\sigma' \left\langle T_\tau c^{\dagger}_{p\sigma}(\vec{k_1}, \tau) c_{q\sigma}(\vec{k_1} + \vec{q}, \tau) c^{\dagger}_{s\sigma'}(\vec{k_2} + \vec{q}, 0) c_{t\sigma'}(\vec{k_2}, 0) \right\rangle, \tag{8}$$

$$\chi^{(s^{+-})pq}_{st}(\vec{q}, \tau) \equiv \frac{1}{N} \sum_{\vec{k_1}\vec{k_2}} \left\langle T_\tau c^{\dagger}_{p\uparrow}(\vec{k_1}, \tau) c_{q\downarrow}(\vec{k_1} + \vec{q}, \tau) c^{\dagger}_{s\downarrow}(\vec{k_2} + \vec{q}, 0) c_{t\uparrow}(\vec{k_2}, 0) \right\rangle, \tag{9}$$

$$\chi^{(s^{-+})pq}_{st}(\vec{q}, \tau) \equiv \frac{1}{N} \sum_{\vec{k_1}\vec{k_2}} \left\langle T_\tau c^{\dagger}_{p\downarrow}(\vec{k_1}, \tau) c_{q\uparrow}(\vec{k_1} + \vec{q}, \tau) c^{\dagger}_{s\uparrow}(\vec{k_2} + \vec{q}, 0) c_{t\downarrow}(\vec{k_2}, 0) \right\rangle. \tag{10}$$

Note that for the noninteracting limit, we have $\chi^{(s^z)} = \chi^{(s^{+-})} = \chi^{(s^{-+})} = \chi^{(s)} = \chi^{(c)} = \chi^{(0)}$. The RPA-renormalized spin and charge susceptibilities can be expressed by Feynman diagrams in Fig. S10, and can be obtained by solving the related Dyson equations

$$\chi^{(s)}(\vec{q}, i\omega_n) = [I - \chi^{(0)}(\vec{q}, i\omega_n) U^{(s)}]^{-1} \chi^{(0)}(\vec{q}, i\omega_n), \tag{11}$$

$$\chi^{(c)}(\vec{q}, i\omega_n) = [I + \chi^{(0)}(\vec{q}, i\omega_n) U^{(c)}]^{-1} \chi^{(0)}(\vec{q}, i\omega_n), \tag{12}$$

where $\chi^{(s)}(\vec{q}, i\omega_n)$, $\chi^{(c)}(\vec{q}, i\omega_n)$ and $U^{(s,c)}$ are treated as 16×16 matrices (the upper or lower two indices are viewed as one number) with elements of the matrix $U^{(s)}$ and $U^{(c)}$ given by

$$U^{(s)pq}_{st} = \begin{cases} U, & p = q = s = t \\ J_H, & p = q \neq s = t \\ J_H, & p = s \neq q = t \\ V, & p = t \neq s = q \end{cases} \tag{13}$$

$$U^{(c)pq}_{st} = \begin{cases} U, & p = q = s = t \\ 2V - J_H, & p = q \neq s = t \\ J_H, & p = s \neq q = t \\ 2J_H - V, & p = t \neq s = q \end{cases} \tag{14}$$

Note that the matrix elements $U^{(s,c)}$ are non-zero only if p, q, s, t are the same layer indices for on-site interactions. There exists a critical interaction strength $U_c^{(s/c)}$ for spin or charge, respectively. When $U > U_c^{(s,c)}$, the denominator matrix in RPA-renormalized spin or charge susceptibility will have zero determinant, i.e., diverge for some wavevectors; therefore, the RPA treatment becomes invalid. The divergence of spin or charge susceptibility for $U > U_c^{(s)}$ or $U > U_c^{(c)}$ implies magnetic order or charge order, respectively. We focus on the region $U \lesssim \min\left[U_c^{(s)}, U_c^{(c)}\right]$, and a Cooper pair $c_t(\vec{q})c_s(-\vec{q})$ can be scattered to $c_p^\dagger(\vec{k})c_q^\dagger(-\vec{k})$ by exchanging spin or charge fluctuations. The effective pairing interaction is given by

$$V_{\text{eff}}^{\text{RPA}} = \frac{1}{N} \sum_{pqst,\vec{k},\vec{q}} \Gamma_{st}^{pq}(k,q) c_p^\dagger(\vec{k}) c_q^\dagger(-\vec{k}) c_s(-\vec{q}) c_t(\vec{q}). \tag{15}$$

In the RPA level, we include both bubble and ladder diagrams in calculating the effective pairing interaction as shown in Fig. S11. In the singlet channel, the effective vertex $\Gamma_{st}^{pq}(k,q)$ is given by,

$$\begin{aligned}\Gamma_{st}^{pq(s)}(\vec{k},\vec{q}) = \Big(\frac{U^{(c)}+3U^{(s)}}{4}\Big)_{st}^{pq} &+ \frac{1}{4}\Big[3U^{(s)}\chi^{(s)}(\vec{k}-\vec{q})U^{(s)} - U^{(c)}\chi^{(c)}(\vec{k}-\vec{q})U^{(c)}\Big]_{qs}^{pt} \\ &+ \frac{1}{4}\Big[3U^{(s)}\chi^{(s)}(\vec{k}+\vec{q})U^{(s)} - U^{(c)}\chi^{(c)}(\vec{k}+\vec{q})U^{(c)}\Big]_{qt}^{ps},\end{aligned} \tag{16}$$

while in the triplet channel, it is

$$\begin{aligned}\Gamma_{st}^{pq(t)}(\vec{k},\vec{q}) = \Big(\frac{U^{(c)}-U^{(s)}}{4}\Big)_{st}^{pq} &- \frac{1}{4}\Big[U^{(s)}\chi^{(s)}(\vec{k}-\vec{q})U^{(s)} + U^{(c)}\chi^{(c)}(\vec{k}-\vec{q})U^{(c)}\Big]_{qs}^{pt} \\ &+ \frac{1}{4}\Big[U^{(s)}\chi^{(s)}(\vec{k}+\vec{q})U^{(s)} + U^{(c)}\chi^{(c)}(\vec{k}+\vec{q})U^{(c)}\Big]_{qt}^{ps},\end{aligned} \tag{17}$$

where the vertex $\Gamma_{st}^{pq(t)}(\vec{k},\vec{q})$ has been symmetrized for the singlet channel and anti-symmetrized for the triplet channel. We only consider the intra-band pairings, and obtain the following effective pairing interaction

$$V_{eff} = \frac{1}{N} \sum_{\alpha\beta,\vec{k},\vec{q}} V^{\alpha\beta}(\vec{k},\vec{q}) c_\alpha^\dagger(\vec{k}) c_\alpha^\dagger(-\vec{k}) c_\beta(-\vec{q}) c_\beta(\vec{q}), \tag{18}$$

where $\alpha, \beta = 1, \cdots, 4$ are band indices, and the effective pairing interaction vertex $V^{\alpha\beta}(\vec{k},\vec{q})$ has the form

$$V^{\alpha\beta}(\vec{k},\vec{q}) = \sum_{pqst} \Gamma_{pq}^{st}(\vec{k},\vec{q}) \xi_p^{\alpha,*}(\vec{k}) \xi_q^{\alpha,*}(-\vec{k}) \xi_s^{\beta}(-\vec{q}) \xi_t^{\beta}(\vec{q}). \tag{19}$$

In the mean-field approximation, the effective pairing interaction can be decoupled as

$$\frac{1}{N} \sum_{\alpha\beta,\vec{k},\vec{q}} V^{\alpha\beta}(\vec{k},\vec{q}) c_\alpha^\dagger(\vec{k}) c_\alpha^\dagger(-\vec{k}) c_\beta(-\vec{q}) c_\beta(\vec{q}) = \sum_{\vec{k},\alpha} \Big(\Delta_{\vec{k}}^\alpha c_\alpha^\dagger(\vec{k}) c_\alpha^\dagger(-\vec{k}) + h.c.\Big), \tag{20}$$

and the self-consistent pairing gap equation can be derived

$$\Delta_{\vec{k}}^{\alpha} = \frac{1}{N} \sum_{\vec{q}\beta} V^{\alpha\beta}(\vec{k}, \vec{q}) \langle c_\beta(-\vec{q}) c_\beta(\vec{q}) \rangle. \tag{21}$$

Near the superconducting critical temperature T_c, we can linearize the self-consistent pairing gap equation as follows

$$\Delta_{\vec{k}}^{\alpha} = -\sum_{\beta\vec{q}} V^{\alpha\beta}(\vec{k}, \vec{q}) \times \frac{\tanh(\frac{\beta_c}{2}|\tilde{\epsilon}_\beta(\vec{q})|)}{|\tilde{\epsilon_\beta}(\vec{q})|} \times \Delta_{\vec{q}}^{\beta}, \tag{22}$$

where $\beta_c = \frac{1}{k_B T_c}$. The solution of the linearized gap equation becomes the standard eigenvalue problem of the effective pairing interaction matrix $V^{\alpha\beta}(\vec{k}, \vec{q})$, which determines T_c and the leading pairing symmetry. $\Delta_{\vec{k}}^{\alpha}$ represents the gap function on the α-th Fermi surface near T_c. The leading pairing symmetry is determined by the largest eigenvalue λ.

SM7. Effect of free relaxation

We emphasize that we simulate the thin film heterostructure with a simple half-UC-thick crystal *using the experimentally determined parameters*. The parameters of lattice constants are taken from XRD, and the ion distances come from the STEM experiments with a sufficiently small error bar ± 0.05Å. The positions of these heavy ions are well determined in STEM than oxygen atoms. Hence, it is reasonable to relax only the oxygen atoms. These distances are maintained by the strain passed from the complicated interface of the substrate.

The relax-all-system by itself is, of course, interesting. However, we should notice that such free relaxation does not respect the experimentally measured distances between ions. The main text still presents all the results using the experimentally determined crystal structure.

Let us check what will happen to the band structure if we relax all the ions. We have performed such a free relaxation and found that the distance between the inter-plane Ni ions drops from 4.28 Å to 3.97 Å. This again suggests that the substrate could maintain such a large inter-layer Ni-Ni distance, which is not considered here. In the following, we systematically compare bands, the tight-binding model, and Fermi surfaces in the DFT+U level, then compare the spectral functions and Fermi surfaces in the CDMFT level.

As shown in Table S3, most tight-binding parameters change slightly by a few percent when all atoms are relaxed. For example, the crystal-field splitting $\epsilon_x - \epsilon_z$ among e_g orbitals is 0.482 eV in the relax-all-system, slightly smaller than its relax-O-only counterpart 0.519 eV. The intra-plane nearest-neighbor hopping t_1^x and t_z^1 change only a few meV. The most sensitive parameter

to free relaxation is $t_\perp^z$ the inter-layer hopping between the d_{z^2} orbitals, which changes from -0.439 eV to -0.551 eV. As shown by the $\uparrow$ in Fig. S12a, enhanced $t_\perp^z$ shifts upward the δ-band (mainly contributed by z_-), removing the δ pocket in the Fermi surface (comparing Fig. S12e and Fig. S12b) in DFT+U level. Furthermore, the γ-band of the relax-all-system becomes more dispersive and less flat than the relax-O-only-system.

In the CDMFT level, the corresponding quasi-particle weights for the relax-all-system are $Z_{z_+} \approx 0.39$, $Z_{z_+} \approx 0.23$, $Z_{x_+} \approx 0.59$, $Z_{x_-} \approx 0.61$, which are relatively less correlated than the relax-O-only system with $Z_{z_+} \approx 0.25$, $Z_{z_+} \approx 0.12$, $Z_{x_+} \approx 0.54$, $Z_{x_-} \approx 0.54$. This indicates that the strain, which maintains a larger inter-layer Ni-Ni distance, helps enhance the correlation strength. Furthermore, the relax-O-only-system has a flatter dispersion in the γ band near the M-point. In the ARPES experiment, the γ-pocket is fainter in the spectral weight, which is consistent with the broad and blurred γ band or pocket shown in Fig. S12c and Fig. S12d of the relax-O-only system. The relax-all-system instead shows a sharper γ-pocket in Fig. S12g.

SM8. Comparison of Fermi surface between thin film and high-pressure bulk

The CDMFT study of the high-pressure bulk has been demonstrated in Fig. 2b in PRL 133, 096002 (2024) by Ryee et al., which also shows that the inter-layer correlation pushes apart the γ-band and δ-band. Especially, the γ pocket is absent in the high-pressure bulk (see Fig. S13d), resulting from two reasons: (1) the inter-layer correlation is stronger in the high-pressure bulk as the inter-layer hopping between d_{z^2} orbitals are larger; (2) the filling in the high pressure bulk is n=1.5 and there is no hole doping (γ pocket is a hole pocket). We performed the CDMFT study of the high-pressure bulk. Our CDMFT at $n = 1.5$ can quantitatively reproduce the FS obtained by Ryee et al, as shown by Fig. S13c. Indeed, it is not proper to compare the FS of the film at $n = 1.3$ (Fig. 3a) to the FS of the high-pressure bulk at $n = 1.5$ (Fig. S13c-d), as they are very different in the pockets and their shapes. It is instead proper to compare FS of both systems at the same filling $n = 1.3$, as shown in Fig. S13a and Fig. S13b. These two FSs are qualitatively similar, showing a γ hole-pocket. The difference is in the size of each pocket. Furthermore, as its clear FS line shows, the high-pressure bulk has more coherent quasi-particles than the thin film.

SM9. Frequency dependence of self-energy

The quasi-particle weights Z is related to frequency dependence of $\mathrm{Re}\Sigma_{\alpha\sigma}(\omega)$ near the zero-frequency: $Z = \left(1 - \frac{\partial \,\mathrm{Re}\,\Sigma(\omega)}{\partial\omega}\Big|_{\omega=0}\right)^{-1}$. The smaller the quasi-particle weight, the larger the slope in $\mathrm{Re}\,\Sigma(\omega)|_{\omega=0}$. As shown by Fig. S14(a-b), the z_- orbital has the largest slope while the $x_\pm$ orbital

has the smallest slope, with $Z_{z_+} \approx 0.25$, $Z_{z_+} \approx 0.12$, $Z_{x_+} \approx 0.54$, $Z_{x_-} \approx 0.54$ (see Fig. 3b).

As expected, as U increases, the frequency dependence of self-energy becomes more evident. This can be directly seen in Fig. S14 by comparing the dashed lines for the weakly correlated system ($U = 2$ eV) and the solid lines for the strongly correlated system ($U = 3.6$ eV). Especially, even at $U = 3.6$ eV, the self-energy of $x_\pm$ orbitals still shows a weaker frequency dependence than that of $z_\pm$ orbitals, indicating a weak coupling nature of $x_\pm$ orbitals. The $z_\pm$ orbitals are much more correlated, as shown by (1) their small quaisi-particle weight (large slopes in $\mathrm{Re}\Sigma_{\alpha\sigma}(\omega)$), (2) their incoherence at high-energy (larger imaginary part $|\mathrm{Im}\Sigma_{\alpha\sigma}(\omega)|$).

As for the asymmetry between z_+ and z_- orbitals, there are three points as follows: (1) They are different in their quasi-particle weights with the z_- orbitals more correlated $Z_{z_+} \approx 0.25$ vs. $Z_{z_-} \approx 0.12$; (2) The asymmetry is more evident in the strong coupling regime; (3) The low-energy quasi-particle is more well-defined in the z_+ orbital than in the z_- orbital at $U = 3.6$, as $\mathrm{Im}\Sigma_{z_+\sigma}(\omega \to 0) \to 0$ while $\mathrm{Im}\Sigma_{z_-\sigma}(\omega \to 0)$ is still finite (see the red solid line for the z_+ orbital and the orange solid line for the z_- orbital in Fig. S14c near $\omega = 0$).

SM10. The reason why z_- orbital has a larger filling at higher U

As one increases U, the d_{z^2} orbitals become more correlated than the $d_{x^2-y^2}$ orbitals. There is a self-doping effect from the $d_{x^2-y^2}$ orbital to the d_{z^2} orbital. As the bonding z_+ orbital is already near half-filling, the self-doped electron prefers to enter the anti-bonding z_- orbital, which is initially far from half-filling. The spectral function of z_- orbital at larger U shows a typical three-peak structure, with the quasi-particle peak and Hubbard bands typical of correlated metal. The increased filling in z_- orbital forms the lower Hubbard band (see Fig. S15b). The upper Hubbard band also forms and extends to much higher energy than the weak coupling part. As U increases, although the energy level of z_- bands shifts to higher energy (the "mass" center of spectral function), the filling of z_- orbital still increases due to the formation of the lower Hubbard band.

1. G. Zhou, W. Lv, H. Wang, Z. Nie, Y. Chen, Y. Li, H. Huang, W. Chen, Y. Sun, Q.-K. Xue, Z. Chen, *Ambient-pressure superconductivity onset above 40 K in bilayer nickelate ultrathin films*, arXiv:2412.16622v1 (2024).

2. Kresse, G. and Hafner, *Ab initio molecular dynamics for liquid metals*, J., Phys. Rev. B **47,** 558 (1993).

3. G. Kresse and J. Furthmüller, *Efficiency of ab-initio total energy calculations for metals and semiconductors using a plane-wave basis set*, Computational Materials Science **6**, 15 (1996).

4. G. Kresse and J. Furthmüller, *Efficient iterative schemes for ab initio total-energy calculations using a plane-wave basis set*, Phys. Rev. B **54**, 11169 (1996).

5. J. P. Perdew, K. Burke, and M. Ernzerhof, *Generalized gradient approximation made simple*, Phys. Rev. Lett. **77**, 3865 (1996).

6. N. E. Bickers and D. J. Scalapino, *Conserving approximations for strongly fluctuating electron systems. I. Formalism and calculational approach*, Ann. Phys. **193**, 206–251 (1989).

7. N. Witt, E. G. C. P. Van Loon, T. Nomoto, R. Arita, and T. O. Wehling, *Efficient fluctuation-exchange approach to low-temperature spin fluctuations and superconductivity: From the Hubbard model to $Na_xCoO_2{\cdot}yH_2O$*, Phys. Rev. B **103**, 205148 (2021).

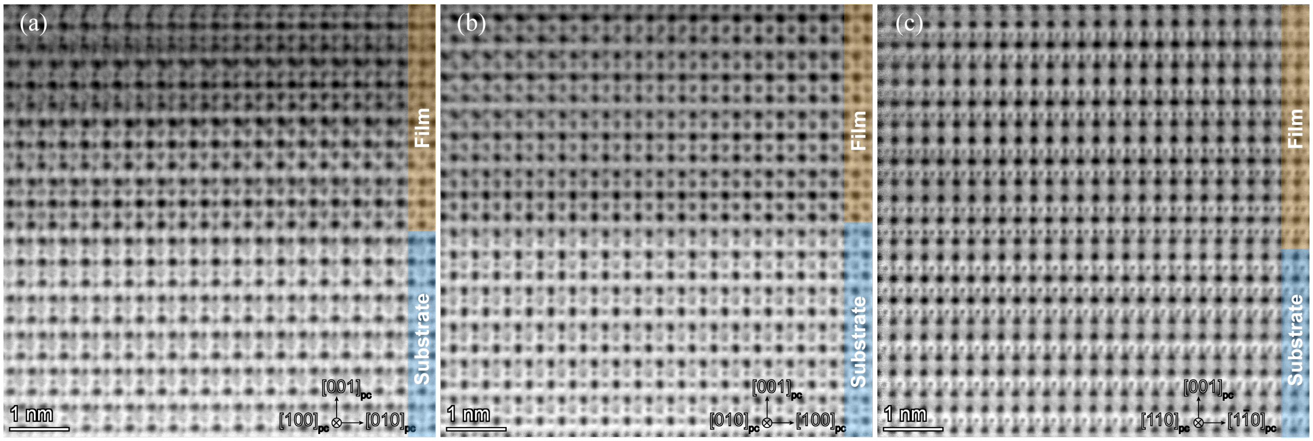

Fig. S1 | Annular bright-field (ABF) images of the cross-section of $La_{2.85}Pr_{0.15}Ni_2O_7$ film. (a-c), The large-scale ABF images, projected along $[100]_p$, $[010]_{pc}$ and $[110]_{pc}$, respectively, delineate the boundaries of the film and the substrate.

Table S1 | The lattice parameters of $La_3Ni_2O_7$ film and substrate $SrLaAlO_4$. STEM measurements are calibrated by the lattice constants obtained from X-ray diffraction experiments. The term "La-La intra" refers to the distance between La atoms in the c-axis direction in the perovskite layer. The term "La-La inter" refers to the distance between adjacent La atoms in the c-axis direction between the perovskite bilayers. All lattice parameters are represented numerically using pseudocube cells.

	**$SrLaAlO_4$**			**$La_3Ni_2O_7$**		
	**cif**	**XRD**	**STEM**	**XRD**	**STEM**	**cif**
**a**	3.7544	3.754	3.75	3.754	-	3.833
**b**	3.7544	3.754	3.75	3.754	-	3.833
**c**	12.649	12.630	12.64	20.758	-	20.518
**Ni-O-Ni angle (°)**	-	-	-	-	180 ± 5	-
**Ni-Ni length**	-	-	-	-	4.04 ± 0.05	-
**La-La intra**	-	-	3.60 ± 0.05	-	3.68 ± 0.05	-
**La-La inter**	-	-	2.75 ± 0.05	-	3.09 ± 0.05	-

Table S2 | Lattice constants and fractional coordinates of non-equivalent atomic positions for the half-UC $La_3Ni_2O_7$ ultra-thin film crystal structure. The Cartesian coordinate of each atom is given by $f_1\mathbf{a} + f_2\mathbf{b} + f_3\mathbf{c}$. In the VCA calculation to simulate Sr-doping, we either dope Sr *only* to the middle La layer with the ratio La:Sr=2:1 in this layer or dope Sr to *all* La layers with the ratio La:Sr=8:1. The crystal structure shown in Fig. 2a can be reproduced with the space group P4/mmm (No. 123; the actual 2D space group is p4/mm). *Make sure that $|c|$ is set 40 Å to get correct spacing between atoms.*

lattice constants			
$\mathbf{a}$(Å)	3.7544	0	0
$\mathbf{b}$(Å)	0	3.7544	0
$\mathbf{c}$(Å)	0	0	40
frac. coord.	f_1	f_2	f_3
La1	0.00000	0.00000	0.40738
La2	0.00000	0.00000	0.50000
Ni1	0.50000	0.50000	0.44651
O1	0.50000	0.50000	0.39523
O2	0.00000	0.50000	0.44793
O3	0.50000	0.50000	0.50000

[!htp]

	$\epsilon_x - \epsilon_z$	t_1^x	t_z^1	$t_\perp^x$	$t_\perp^z$	t_3^{xz}
relax-O-only	0.519	-0.466	-0.126	0.001	-0.439	0.229
relax-all	0.482	-0.463	-0.132	0.005	-0.551	0.232
High-P bulk	0.367	-0.483	-0.110	0.005	-0.635	0.239

Table S3 Comparison of the main tight-binding model parameters between the relax-O-only-system and relax-all-system. We also place the corresponding model parameters of the high-pressure bulk system. The energy is in units of eV .

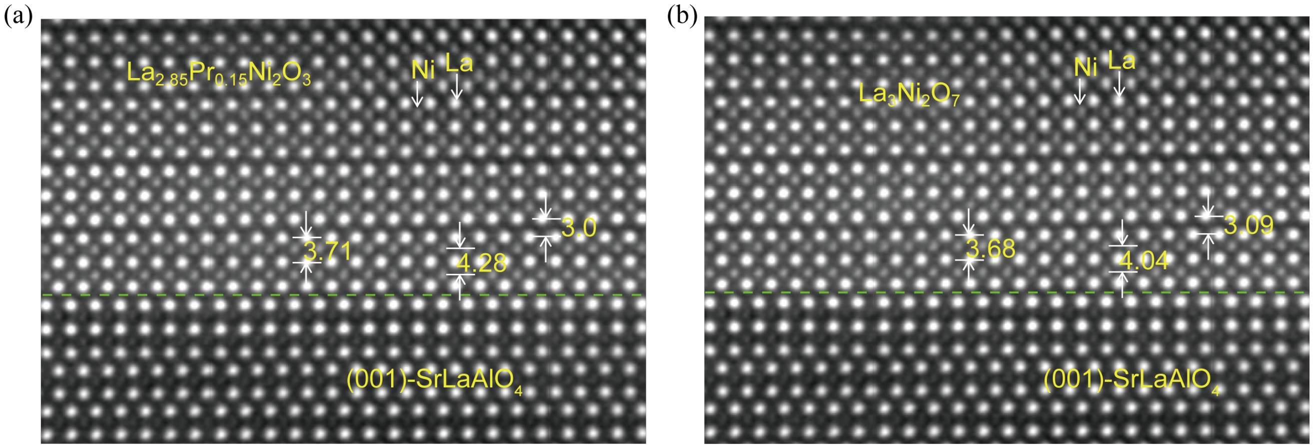

Fig. S2 | High angle Annular Dark Field (HAADF) images with atomic spacings indicated. (a), Atomic spacing of $La_{2.85}Pr_{0.15}Ni_2O_7$ film on $SrLaAlO_4$ substrate. (b), Atomic spacing of the $La_3Ni_2O_7$ film on $SrLaAlO_4$ substrate. The atom spacings in the substrates and the films are calibrated using the XRD measurements.

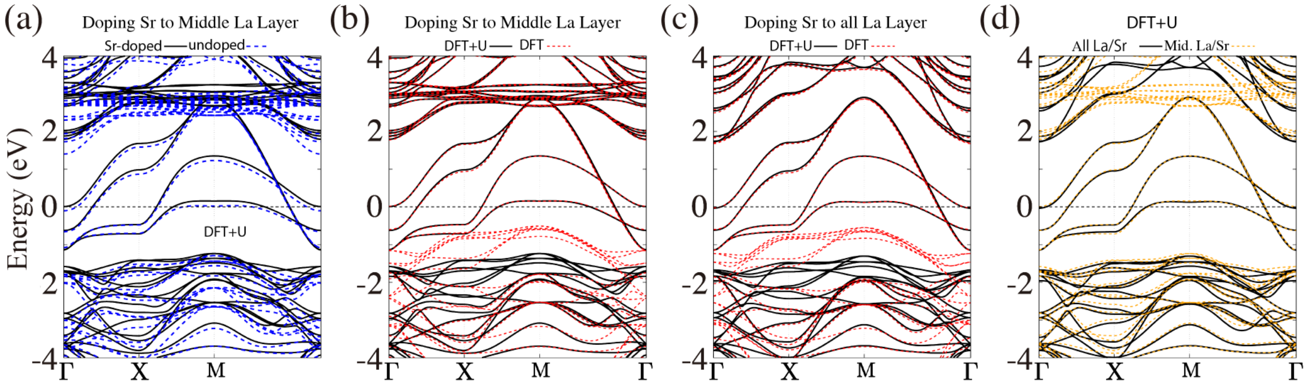

Fig. S3 | Band structures of half-UC ultra-thin film calculated under several indicated conditions. All the bands (black line in (a) and all lines in (b-d)) correspond to a filling of $n \approx 1.33$ (1/6 hole) in Ni-e_g orbitals, except the blue dashed line in (a), corresponding to the Sr un-doped system. $U = 5$ and J =1 eV are used 1in all the DFT+U calculations. (a) Comparison between DFT+U bands of Sr-doped and of Sr-undoped systems when Sr is doped only to the middle layer of La in the ultra-thin film with the composition ratio La:Sr=2:1 in the middle layer (8:1 in total when counting all La over Sr). (b) Comparison between DFT+U bands (black solid line) and DFT bands (red dashed line) when Sr is doped only to the middle La layer. (c) Comparison between DFT+U bands (black solid line) and DFT bands (red dashed line) when Sr is uniformly doped to all La layers with the composition ratio 8:1. (d) Comparison between DFT+U bands of uniform Sr doping (black solid line) and of doping Sr only in the middle layer (orange dashed line).

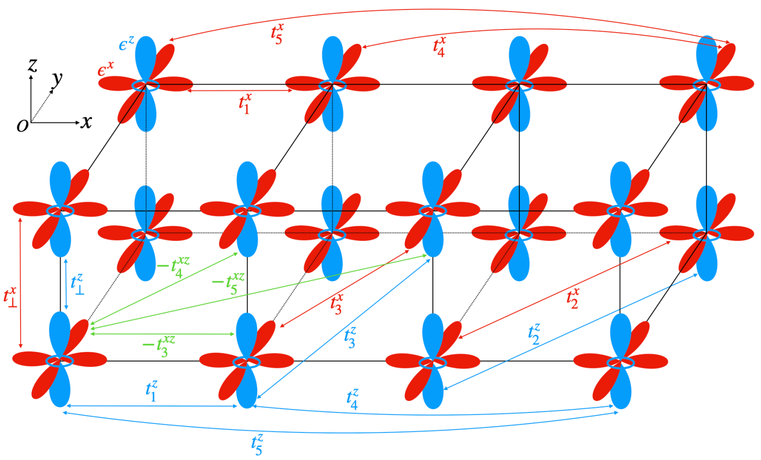

Fig. S4 | Schematic illustration for all parameters of the tight-binding model.

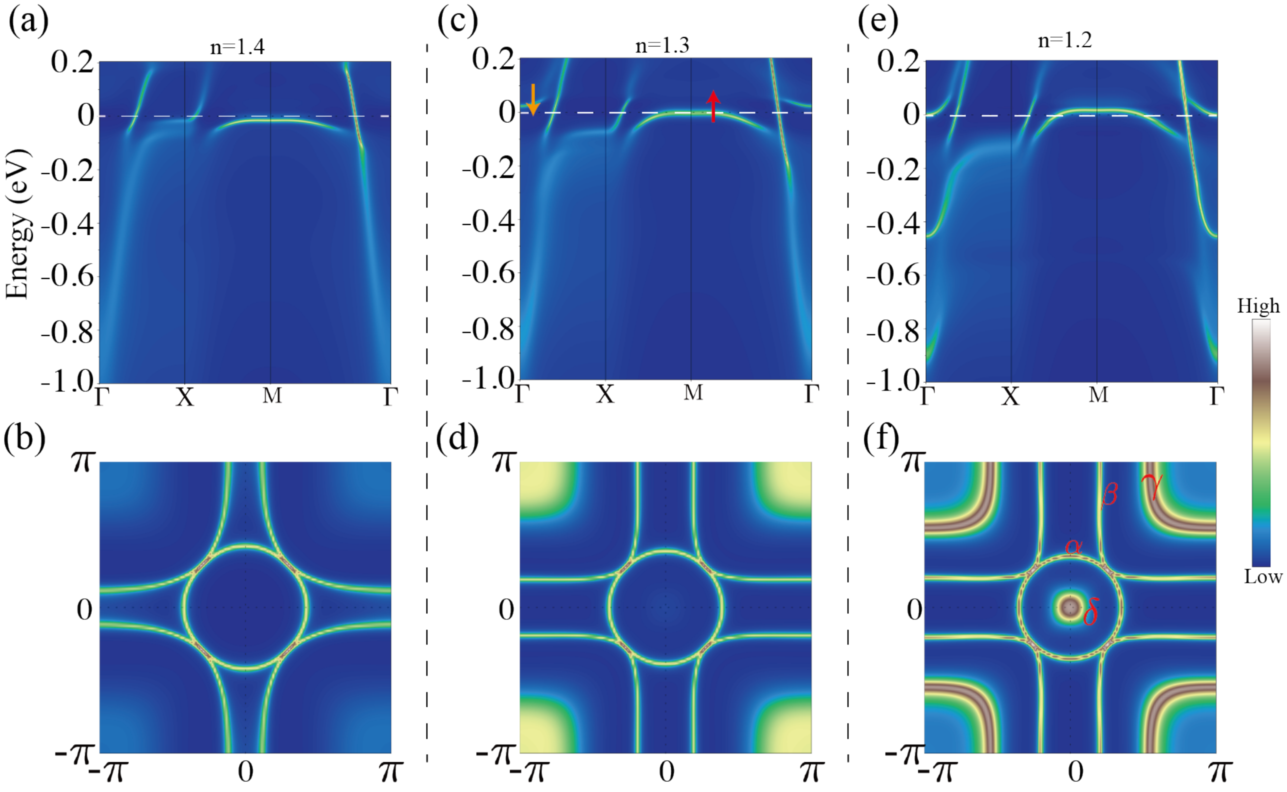

Fig. S5 | Filling n-dependence of $A(\mathbf{k},\omega)$ (top panels) and FS (lower panels) calculated at U =3.6 eV, J =0.56. In all panels, U = 3.6 eV, J=0.56 eV, T =200K.

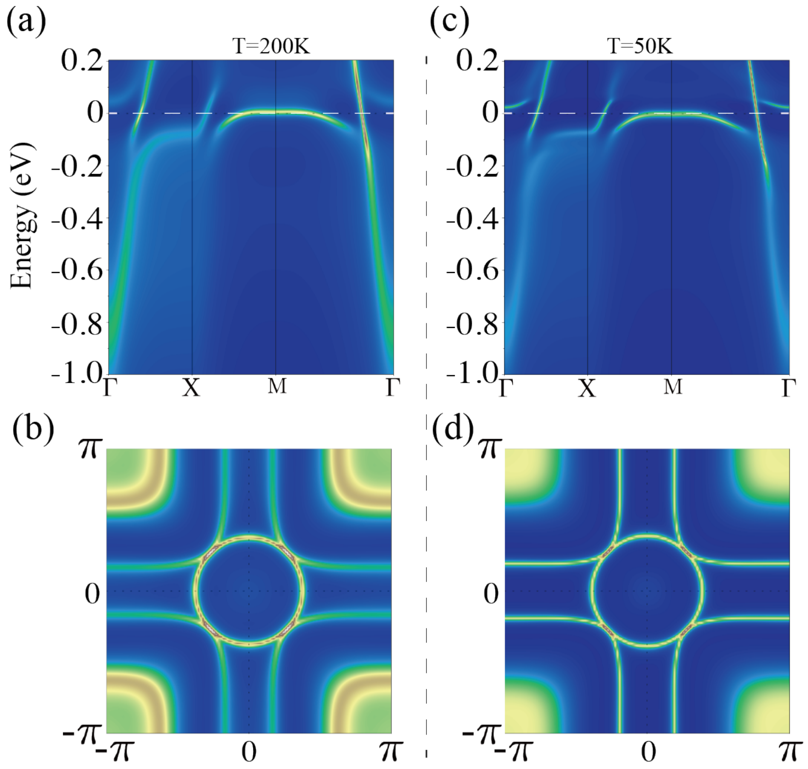

Fig. S6 | Temperature dependence of $A(\mathbf{k},\omega)$ (top panels) and FS (lower panels) calculated in CDMFT at $U = 3.6$ eV, J=0.56 eV, n=1.3. (a-b) T=200 K; (c-d) T=50K.

.

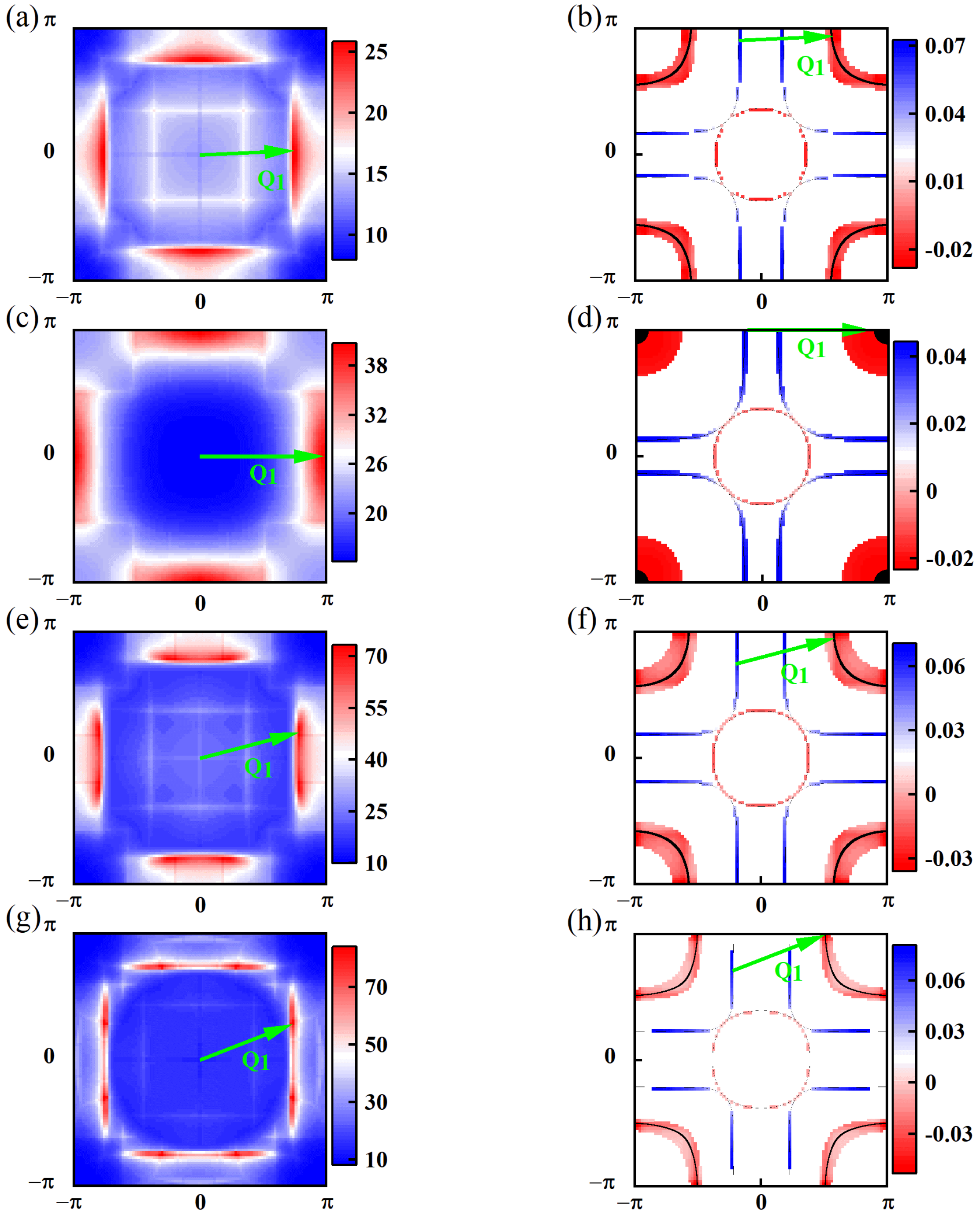

Fig. S7 | The spin susceptibilities and corresponding gap functions on the FS. (a,b) $n = 1.25$, $U = 3.6$ eV and $U_{\text{eff}} = 0.17$eV. (c,d) $n = 1.3$, $U = 3.77$ eV and $U_{\text{eff}} = 0.14$eV. (e,f) $n = 1.3$, $U = 3.5$ eV and $U_{\text{eff}} = 0.205$eV. (g, h) $n = 1.3$, $U = 3$ eV and $U_{\text{eff}} = 0.312$eV.

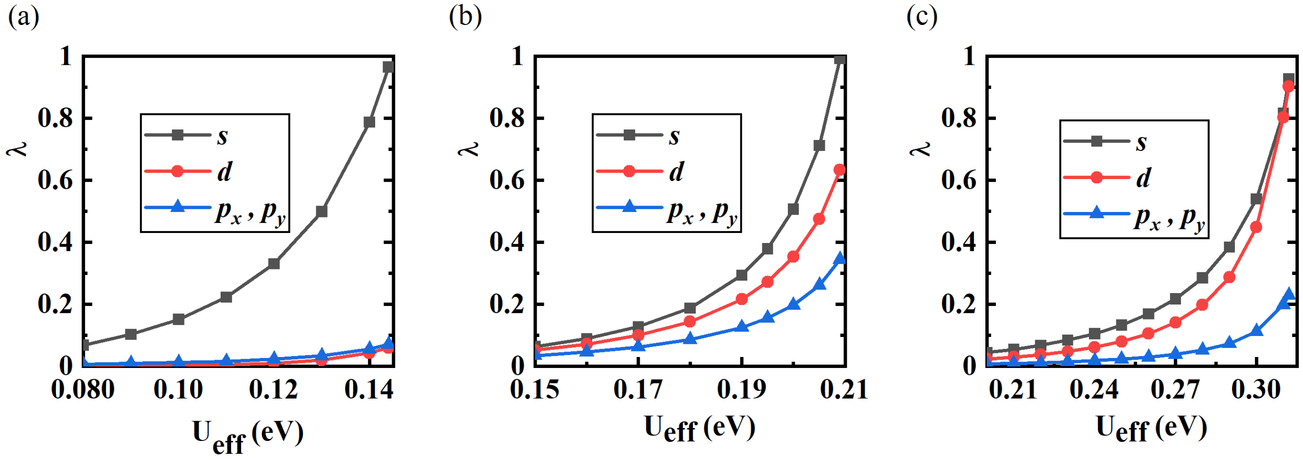

Fig. S8 | The dependence of λ on U_{eff}. (a) $U = 3.77$ eV. (b) $U = 3.5$ eV. (c)$U = 3$ eV. The filling is $n = 1.3$.

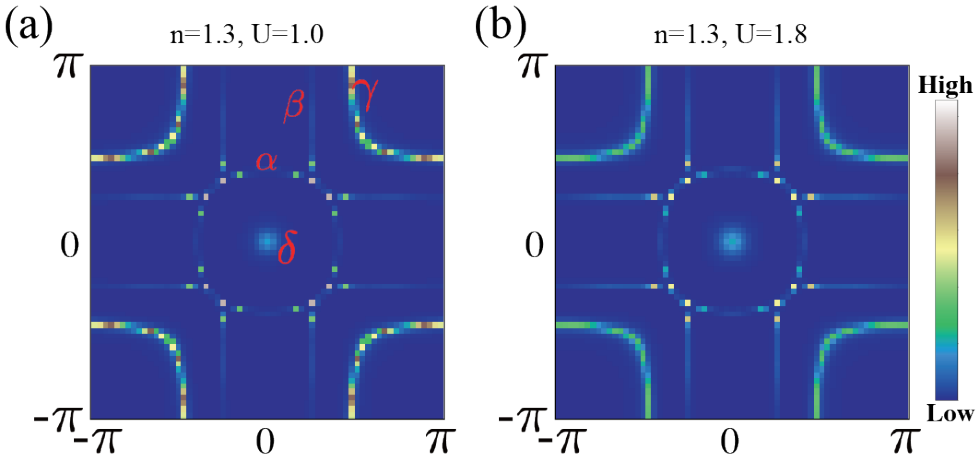

Fig. S9 | Fermi surfaces calculated within FLEX at indicated U and n. The temperature is T =0.001 eV ($\sim$ 11.6K), $J = U/6$,

.

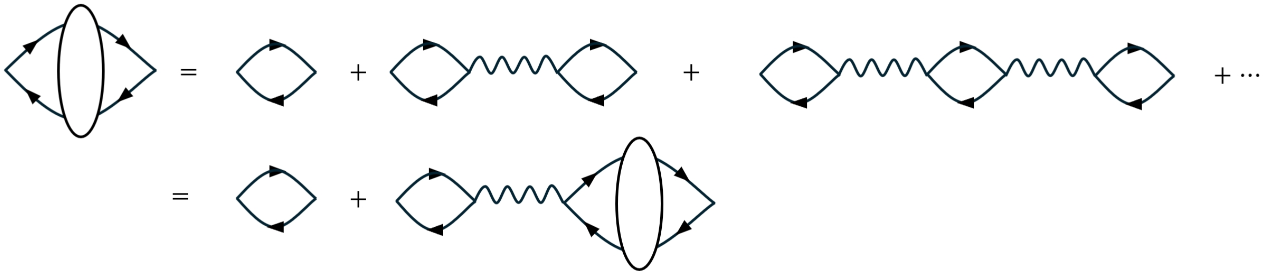

Fig. S10 | Feynman diagrams for the RPA-renormalized susceptibilities.

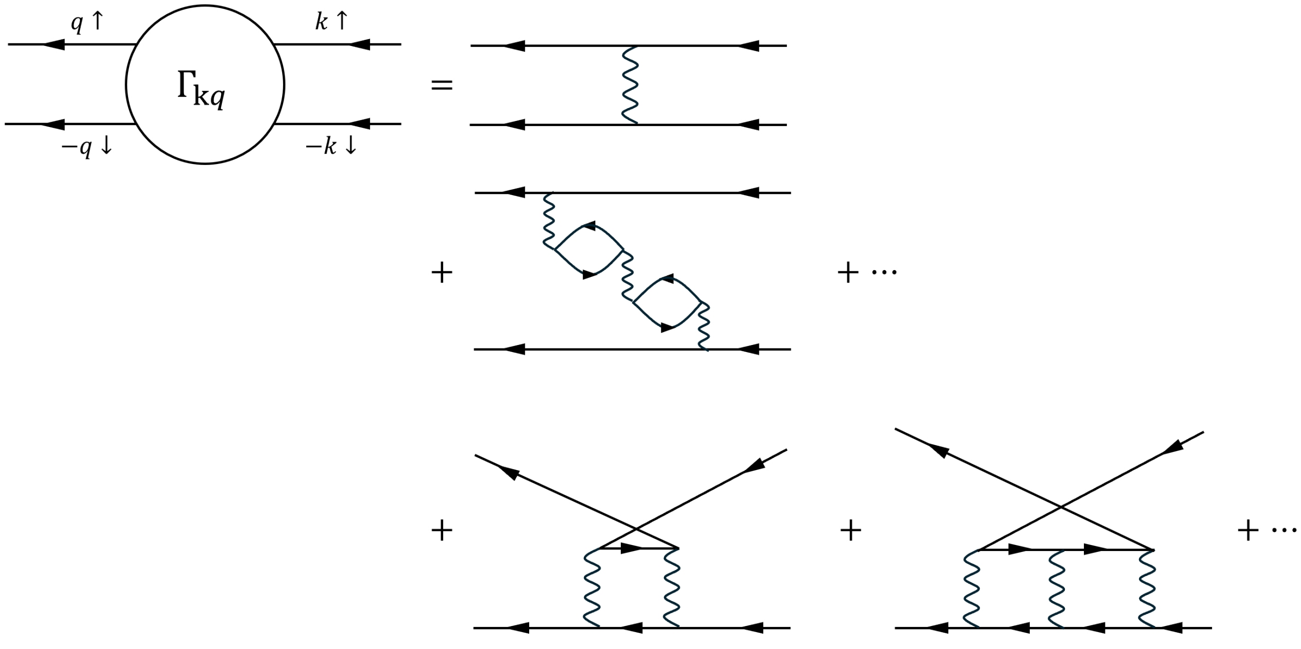

Fig. S11 | Feynman diagrams for the effective pairing interaction for bubble and diagram contributions.

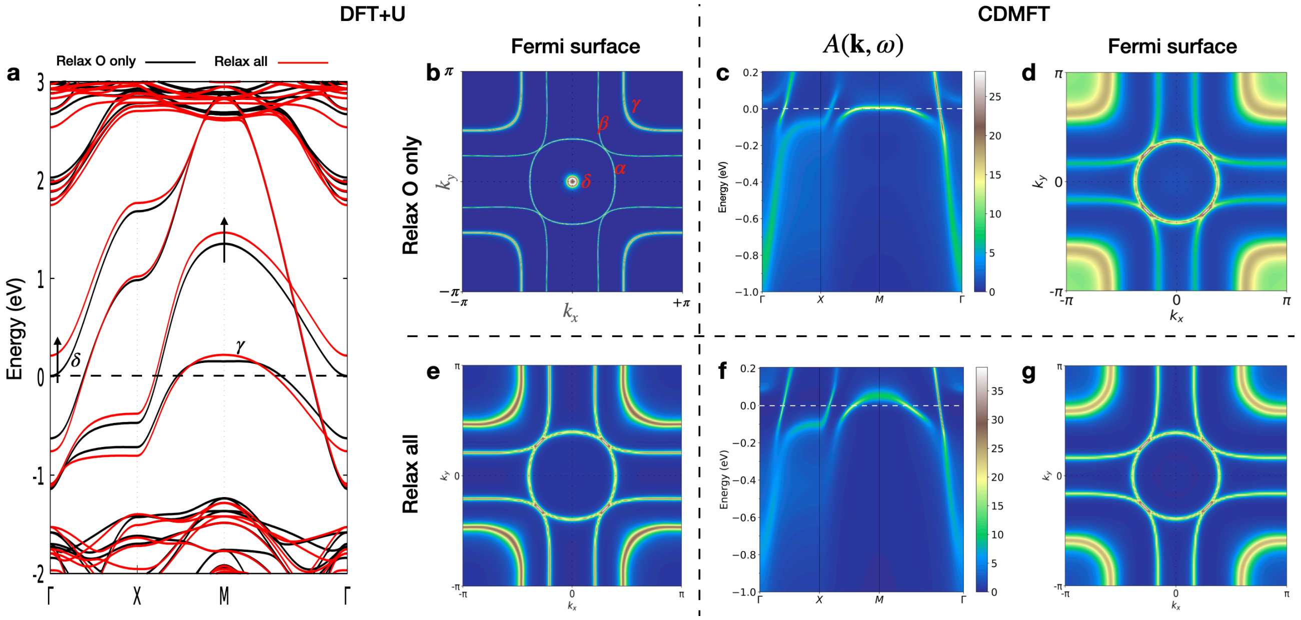

Fig. S12 Comparison of bands, Fermi surface, $A(\boldsymbol{k}, \omega)$ between the relax-O-only system and the relax-all system. The parameters are $T = 200$ K, U =3.6 eV, $J = 0.56$ eV, $n = 1.3$. The panels a, b, and e correspond to DFT+U, while c, d, f, and g correspond to CDMFT.

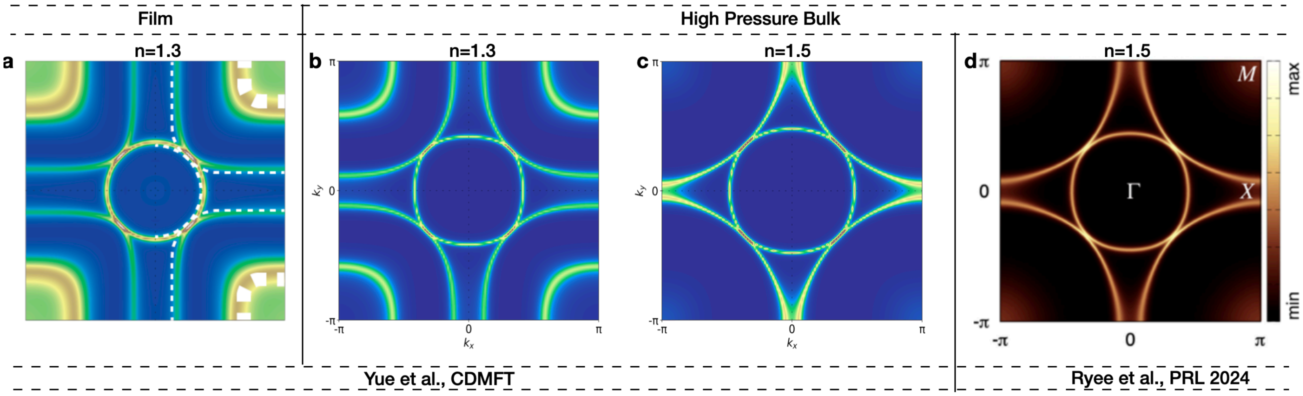

Fig. S13 Comparison of the thin film's Fermi surface (FS) and the high-pressure bulk's FS. Panel a: FS of the thin film. Panel b-d FS of high-pressure bulk at indicated fillings. The fillings are indicated above each panel. Panels a-c are obtained by the authors using a home-made CDMFT. The corresponding tight-binding model Hamiltonian used in (b-c) of the high-pressure bulk is obtained from Luo et al. PRL 131, 126001 (2023). The panel d is reproduced by Fig. 2b of Ryee et al., PRL 133, 096002 (2024). In a-c, the parameters are $T = 200$ K, $U = 3.6$ eV, $J = 0.56$ eV.

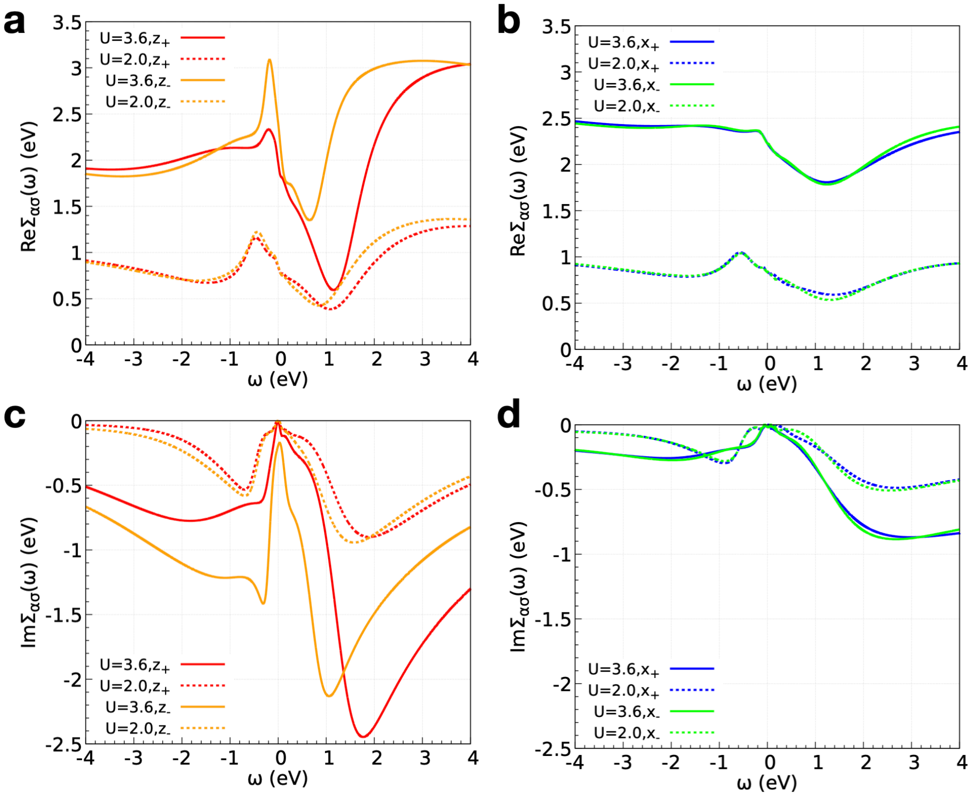

Fig. S14 Comparison of real-frequency self-energy between system at $U = 2.0$ eV (dahsed lines) and system at $U = 3.6$ eV (solid lines). In both panels, T =200 K , J=0.56 eV, n=1.3. Panel a and b shows the real part $\mathrm{Re}\Sigma_{\alpha\sigma}(\omega)$, while c and d the imaginary part $\mathrm{Im}\Sigma_{\alpha\sigma}(\omega)$.

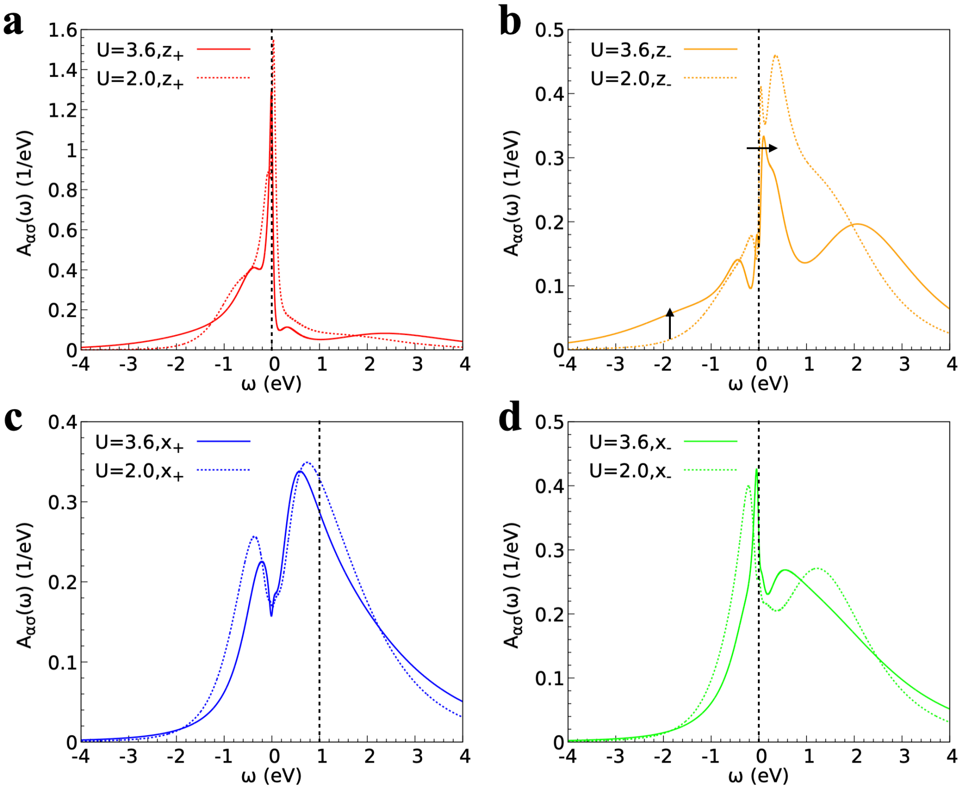

Fig. S15 Comparison of spectral functions $A_{\alpha\sigma}(\omega)$ of all spin-orbitals (panel a for z_+, b for z_-, c for x_+, and d for x_-) between $U = 2$ eV (dashed-line) and $U = 3.6$ eV (solid line). In all panels, $J = 0.56$ eV, $n = 1.3$ and $T = 200$ K. The $\uparrow$ in panel b indicates the appearance of the lower Hubbard band in the z_- orbital as one increases $U = 2$ to $U = 3.6$ eV. The $\rightarrow$ in panel b indicates the upward shift of the energy level of the z_- orbital.
